# A Review of Fault Diagnosis Methods: From Traditional Machine Learning to Large Language Model Fusion Paradigm

**DOI:** 10.3390/s26020702

**Published:** 2026-01-21

**Authors:** Qingwei Nie, Junsai Geng, Changchun Liu

**Affiliations:** 1 School of Mechanical Engineering, Yangzhou University, Yangzhou 225000, China; d.nie@yzu.edu.cn (Q.N.); mz120240894@stu.yzu.edu.cn (J.G.); 2 College of Mechanical and Electrical Engineering, Nanjing University of Aeronautics and Astronautics, Nanjing 210016, China

**Keywords:** fault diagnosis, traditional machine learning, digital twin, knowledge graph, large language model, artificial intelligence

## Abstract

Fault diagnosis is a core technology ensuring the safe and efficient operation of industrial systems. A paradigm shift has been observed wherein traditional signal analysis has been replaced by intelligent, algorithm-driven approaches. In recent years, large language models, digital twins, and knowledge graphs have been introduced. A new stage of intelligent integration has been reached that is characterized by data-driven methods, knowledge guidance, and physical–virtual fusion. In the present paper, the evolutionary context of fault diagnosis technologies was systematically reviewed, with a focus on the theoretical methods and application practices of traditional machine learning, digital twins, knowledge graphs, and large language models. First, the research background, core objectives, and development history of fault diagnosis were described. Second, the principles, industrial applications, and limitations of supervised and unsupervised learning were analyzed. Third, innovative uses were examined involving physical–virtual mapping in digital twins, knowledge modeling in knowledge graphs, and feature learning in large language models. Subsequently, a multi-dimensional comparison framework was constructed to analyze the performance indicators, applicable scenarios, and collaborative potential of different technologies. Finally, the key challenges faced in the current fault diagnosis field were summarized. These included data quality, model generalization, and knowledge reuse. Future directions driven by the fusion of large language models, digital twins, and knowledge graphs were also outlined. A comprehensive technical map was established for fault diagnosis researchers, as well as an up-to-date reference. Theoretical innovation and engineering deployment of intelligent fault diagnosis are intended to be supported.

## 1. Introduction

### 1.1. Research Background and Significance

As global industrial systems shift toward intelligence, connectivity, and high-end development, equipment complexity continues to increase. Higher structural complexity, stronger functional integration, and more diverse operating conditions have been observed. In intelligent manufacturing, energy and power, and aerospace, system coupling has intensified as equipment has become more complex. As such, once local components experience abnormalities or performance degradation, faults are not only concealed and sudden but may also trigger chain reactions rapidly through system coupling relationships. This leads to complete equipment shutdown, production interruption, and even serious safety accidents, thereby resulting in huge economic losses and significant safety risks. For these reasons, the development of efficient, reliable, and intelligent fault diagnosis systems is an urgent engineering requirement [1]. Prior review studies have indicated that intelligent fault diagnosis has become a key enabling technology. Maintenance costs can be reduced, unplanned downtime can be avoided, and system life and reliability can be improved in applications such as rotating machinery, robots, and machine tools [2]. Traditional fault diagnosis has mainly relied on expert experience, mechanism-based analysis, and hand-crafted features derived from signal processing. Good performance is often achieved under low-dimensional and stable operating conditions. Yet, under operating-condition drift, nonlinear noise, and concurrent multi-fault scenarios, feature degradation and diagnostic delay are frequently observed [3,4]. Data-driven intelligent diagnosis has gradually become the mainstream with the improvement of the Industrial Internet of Things and online monitoring systems. Although deep learning enables automatic feature extraction and high-accuracy classification in end-to-end frameworks, strong dependence on large-scale, identically distributed labeled data remains. In practical industrial settings, “few-shot,” weakly annotated, and distribution-shifted conditions are common, and performance bottlenecks are therefore encountered [5]. In recent years, to alleviate the distribution difference problem across multiple operating conditions/pieces of equipment, a large number of studies have focused on transfer learning, domain adaptation, and domain generalization. Nevertheless, negative transfer, class imbalance, and cross-domain alignment difficulties have not been completely solved. In particular, when labeled data in the target domain are scarce or unavailable, diagnostic stability and generalization remain limited [6].

To address few-shot learning and cross-domain generalization, deep transfer diagnosis has been developed along several technical routes. One route is based on discriminative domain alignment across machines and operating conditions. Domain-invariant representations are learned, and improved separability in the target domain is thereby promoted. A second route adopts meta-learning and few-shot strategies. Stronger task-transfer capability is obtained under a “few target samples–rapid adaptation” paradigm. A third route introduces federated transfer learning. Knowledge sharing across enterprises and sites is enabled under constraints imposed by data privacy and data silos [7]. Relevant studies have verified their effectiveness in scenarios such as rolling bearings, gearboxes, pumps, and key nuclear power components. However, strong dependence on source-domain priors is still observed, and robustness remains limited under realistic industrial noise and abrupt operating-condition changes [8].

Digital twins offer a potential path to mitigate the scarcity of real fault data. A high-fidelity virtual counterpart is constructed and updated in real time. Bidirectional mapping and closed-loop interaction between the physical asset and the virtual model are thereby enabled. Various fault modes and degradation trajectories can then be reproduced safely and at low cost in the virtual space. Controllable prior data and mechanism-based constraints can therefore be supplied for intelligent diagnosis [9,10]. Prior studies have shown consistent benefits under limited fault samples and severe operating-condition variation. Source-domain training sets can be synthesized through physical–virtual fusion. Transfer pre-training and adversarial alignment can also be supported. As a result, few-shot diagnosis and cross-domain adaptation performance can be improved. In parallel, digital-twin-driven predictive maintenance has progressed from proof-of-concept to multi-industry deployment. The transition from post-event identification to pre-event prediction has been facilitated. Nevertheless, digital twins still face challenges such as high-precision physical modeling, real-time data synchronization, and cross-platform integration, resulting in substantial modeling costs, and the cost of multi-physics coupling modeling for complex equipment is 3–5 times higher than that for single components. For these reasons, deeper coordination with data-driven methods is still required.

At the same time, knowledge graphs provide a complementary capability. Structured representation and interpretable reasoning over complex fault mechanisms are supported. Industrial faults often propagate through causal chains and exhibit multi-component correlations. Diagnostic credibility and traceability are therefore difficult to guarantee when only black-box models are used. In knowledge graphs, fault phenomena, operating conditions, component topology, and expert knowledge are encoded into computable semantic networks via ontologies and triples. Cross-equipment knowledge reuse is enabled. Integration with graph neural networks and rule-based reasoning can also be achieved, which supports propagation mechanism learning and root-cause localization [11]. In recent years, research on knowledge graph construction, embedding learning, and reasoning diagnosis for scenarios such as industrial process systems, additive manufacturing, and robots has grown rapidly. Strong interpretability has been demonstrated, and high potential for engineering adaptation has been indicated. However, large-scale industrial deployment is still constrained. High-quality fault corpora are scarce. Fine-grained entity and relation extraction remains difficult. Temporal evolution is often modeled insufficiently. As a result, the broad application of knowledge graphs to industrial fault diagnosis remains limited.

Further, large language models and their multimodal variants are reshaping fault diagnosis paradigms. Traditional deep models are typically trained for a single scenario, and “one model per scenario” is often assumed. In contrast, large language models leverage extensive semantic knowledge and strong reasoning capacity. Unified representations can be learned across heterogeneous sources, including text, logs, maintenance records, and sensor signals. Cross-task transfer can then be supported. Zero-shot and few-shot diagnosis can be enabled through prompt-based learning, retrieval-augmented generation, or lightweight fine-tuning. Recent studies have shown that large model-based diagnostic frameworks for complex equipment can improve fault identification accuracy. Improvements in human–machine collaboration efficiency have also been observed. Distinct advantages have been demonstrated in knowledge-intensive stages, such as diagnostic explanation and maintenance recommendation generation. In parallel, complementarity between large language models and knowledge graphs has been emphasized. Knowledge graphs can be used to mitigate scarcity of industrial domain knowledge and to impose controllability constraints. Large language models can, in turn, enhance knowledge graph construction and semantic reasoning. A closed-loop intelligent diagnosis scheme can thus be formed through “data–knowledge–model” collaboration. Large language models are still in the early exploration stage in industrial fault diagnosis (the alignment of temporal signals and language modalities and safe and reliable training in industrial-specific domains remain to be solved). Nevertheless, their deep integration with digital twins, knowledge graphs, and traditional machine learning has been recognized as a critical direction for the next generation of highly reliable fault diagnosis.

In summary, the high-risk operating characteristics and real-world data constraints of complex industrial systems require a shift in fault diagnosis. Isolated data-driven or mechanism-driven methods are no longer sufficient. An integrated technical framework is required, in which digital twins provide physical–virtual mapping and controllable simulation, knowledge graphs provide structured knowledge representation and interpretable reasoning, and large language models provide cross-modal generalization and intelligent interaction. Under this framework, collaboration mechanisms among these three technologies and conventional machine learning and deep learning should be investigated. Key bottlenecks in few-shot learning and cross-domain generalization can be mitigated. Diagnostic credibility and transferability can be strengthened. At the system level, the transition from passive maintenance to predictive maintenance and intelligent decision-making can be accelerated, with substantial engineering value and clear academic significance.

### 1.2. Division of Technological Evolution Stages

The development of fault diagnosis technology can be divided into four stages: the traditional stage (based on signal processing and rule-based reasoning, such as Fourier transform and expert systems), the machine learning stage (driven by supervised/unsupervised learning to realize the mapping between features and faults), the digital twin and knowledge graph-assisted stage (physical–virtual fusion and knowledge guidance to improve diagnostic interpretability), and the intelligent fusion stage (deep collaboration of data, knowledge, and physical–virtual models empowered by large language models). As shown in Table 1, a technical characteristic comparison table is incorporated, covering four types of technologies (i.e., traditional machine learning (supervised/unsupervised), digital twins, knowledge graphs, and large language models).

**Figure 1:**
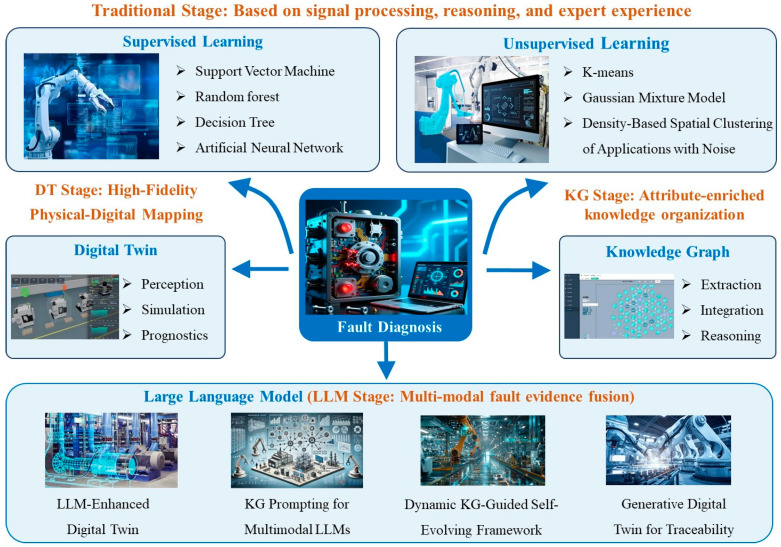
Division of Technological Evolution Stages of Fault Diagnosis.

Across these stages, technologies have progressed in a cumulative manner rather than through replacement. Fault diagnosis has therefore been advanced toward higher precision, stronger real-time capability, improved predictability, and increased interpretability. The detailed stage division is provided in Figure 1.

In the traditional stage, fault diagnosis is mainly based on signal processing and rule-based reasoning. Time-domain, frequency-domain, and time–frequency features are extracted. Methods such as the Fourier transform [12,13,14], wavelet transform [15,16,17], and empirical mode decomposition [18,19,20] are typically used. Fault identification is then performed by combining these features with expert systems or mechanism-derived rules. When operating conditions are relatively stable and fault modes are limited, these methods offer clear advantages. Implementation is straightforward and interpretability is strong. However, limitations have become increasingly evident as dimensionality increases and operating conditions become more complex. Strong reliance on expert experience and prior mechanism knowledge is still required. Many studies have shown that under strong noise, operating-condition drift, and concurrent multi-fault settings, handcrafted feature engineering often fails to capture distributional changes adequately. Diagnostic performance is therefore degraded substantially. This limitation has provided the motivation for subsequent data-driven approaches [2].

As industrial systems become increasingly complex and interconnected, the challenges of identifying and diagnosing faults have become more severe. Traditional diagnostic methods rely heavily on fixed models or expert-driven rules. In real operating environments, these assumptions are frequently violated. Performance is therefore degraded under dynamic conditions and unpredictable disturbances. This limitation has driven the adoption of more adaptive, data-driven approaches. Machine learning (ML) and deep learning (DL) have been considered particularly promising. In this stage, traditional signal processing methods such as Fourier transform and wavelet analysis are still widely used as feature preprocessing steps in supervised/unsupervised learning models, forming a hybrid workflow of signal processing and data modeling. Data can be learned directly, and faults can be detected and classified without explicit model specification [21,22,23,24,25,26,27]. Supervised learning is a commonly used machine learning method. Its typical approach is to first extract features using signal processing or statistical methods, and then use classifiers such as artificial neural networks (ANNs) [28,29], support vector machines (SVMs) [30,31,32], random forests (RFs) [33,34], and k-nearest neighbors (kNN) [35,36] to learn the mapping between features and fault types. Thus, this method is particularly effective when large datasets containing clearly labeled fault examples can be obtained [24,25,26,27].

However, in practice, large-scale labeled fault datasets are often unavailable. In such settings, unsupervised learning provides essential support for industrial scenarios with no labels or only weak labels. Representative methods include clustering algorithms such as k-means [36], GMM [37], and DBSCAN [38]. Cluster analysis (CA) is a statistical-based strategy. After clustering data with similar features, multivariate analysis can be used to leverage the correlation between variables for fault detection and diagnosis [36]. In recent years, autoencoders and their variants have also been adopted. Unsupervised feature learning is performed, and anomalies are identified through reconstruction errors in complex nonlinear processes [39]. In addition, recent work has indicated that for rotating machinery and unstable operating conditions, deep unsupervised and self-supervised models are becoming effective under weak annotation constraints [40]. Technologies such as clustering and anomaly detection enable the system to identify abnormal behaviors or deviations from normal patterns, providing valuable insights even without explicit fault labels [23,24,25,26,27].

With advances in deep learning, convolutional neural networks (CNNs), recurrent neural networks (RNNs), graph neural networks (GNNs), deep neural networks (DNNs), and deep belief networks (DBNs) have been widely applied to end-to-end fault identification using vibration signals and multi-sensor data [41,42,43,44,45]. Feature representation capability has been strengthened, and diagnostic accuracy has been improved substantially in many studies [46]. However, deep learning also has some limitations that hinder its further development, progress, and application in complex real-world scenarios. Deep learning is strongly dependent on large-scale labeled training datasets. When labels are scarce, overfitting is often induced and generalization is weakened. Moreover, strict assumptions about the consistency between training and test distributions are typically imposed. When models are trained on distributions that differ from those of the target domain, performance degradation can be pronounced under distribution shift. Transfer learning is another branch of machine learning. The core idea is analogous to human learning from prior experience with limited examples. Improved learning performance can be achieved under sparse training data, and generalization across related yet distinct scenarios can be enhanced [47]. Combined with transfer learning, it is used for fault diagnosis across operating conditions and equipment, alleviating the performance degradation caused by distribution shift to a certain extent [48].

Traditional data-driven fault diagnosis depends on the availability of sufficient measured data. In several critical industrial settings, such data cannot be obtained reliably. The deployment of purely data-driven diagnosis is therefore constrained in many engineering applications [49,50]. Digital twins address this limitation by coupling physical systems with virtual models. Artificial intelligence algorithms are integrated, and multi-source data are used for real-time model updating. Real-time monitoring can be achieved. Future equipment states can also be simulated and predicted, often with high efficiency and accuracy. They have gained extensive attention in engineering and academic fields such as aerospace [51], industry [50], energy [52], power grids [53], automobiles [54,55,56], transportation [57], and intelligent manufacturing [58,59,60]. Recent reviews have reported multiple fault-diagnosis use cases. Fault feature visualization, virtual sensor construction, and digital sample enhancement have been demonstrated in rotating machinery and motor systems. Diagnostic accuracy and remaining-life prediction robustness have been improved accordingly [61]. By constructing high-fidelity virtual models, equipment behavior can be simulated under diverse operating conditions. The generated data can approximate real outputs under both normal and fault states [62]. Using these fault data, the equipment performance can be analyzed and evaluated more in-depth. Nevertheless, several challenges persist. Modeling cost remains high in many applications. Cross-platform interoperability is still limited. Long-term physical–virtual consistency is difficult to maintain. Hybrid architectures have therefore been adopted in many studies. A “physical model + deep-network compensation + data assimilation” scheme is commonly used. Transfer learning or adversarial alignment is then applied to transfer knowledge from the twin space into diagnosis models operating on real data, which supports knowledge sharing and performance improvement across physical and virtual domains [10]. Overall, this stage does not deviate from the core framework of machine learning. And it provides additional training samples for the model through the fusion of virtual and real data, which addresses the black-box problem of machine learning via knowledge modeling.

In the manufacturing field, the accuracy of knowledge services is crucial [63,64]. By converting a large amount of data into structured and operable knowledge, knowledge graphs have become an indispensable tool for improving the accuracy of knowledge services [65]. In industrial systems, faults often propagate in the form of causal chains along equipment topology and process flows. Knowledge graphs organize heterogeneous data into a structured, human-readable, and machine-operable form, which supports intelligent decision-making and automated operations. Consequently, knowledge-based fault diagnosis methods are inherently interpretable. Complex mathematical mechanism models are not necessarily required. In addition, diagnostic knowledge can be updated and expanded in a continuous manner as new data and expert information become available. On this basis, knowledge graph embeddings, graph neural networks, and logical rules can be combined. Fault propagation paths can then be inferred, and root causes can be localized. Cross-equipment and cross-project knowledge reuse can also be supported. Studies on industrial equipment have shown that knowledge graph-based methods can explicitly characterize the temporal and dependency relationships between fault events, and have obvious advantages over pure data-driven methods in terms of interpretability and diagnostic traceability. However, knowledge graphs remain underutilized in fault diagnosis. High-quality graph construction is also challenging. Ontology definition is often difficult. Fine-grained knowledge extraction is particularly demanding, especially when nested entities are present. When a high-quality knowledge graph is built, fault diagnosis can be strengthened substantially. Fault patterns can be identified. Potential faults can be predicted. Maintenance plans can be optimized. More efficient and effective fault management can then be achieved. In practice, ontology construction still depends heavily on expert experience [66]. Expert knowledge has limitations and subjectivity, and the views of different experts may complicate the ontology construction process. At the same time, due to concerns about technical privacy, industrial enterprises are unwilling to share fault diagnosis corpus resources. This hinders the development of more advanced knowledge graphs. Moreover, many existing corpora contain complex nested entities and exhibit severe class imbalance. Named entity recognition is consequently made difficult [65]. Therefore, these challenges greatly hinder the construction of knowledge graphs, thereby affecting the maintenance plan arrangement and root cause determination after fault diagnosis. Large language models have achieved strong performance across many natural language processing tasks. Increasing interest has therefore been generated in industrial settings. Recent work has explored the use of large language models to support fine-grained equipment-fault knowledge graph construction. Automated entity–relation extraction and graph completion have been applied. Data scarcity in industrial fault corpora has been partially alleviated, and a foundation has been established for subsequent knowledge-enhanced diagnosis [67].

The emergence of large language models (LLMs) has enabled task-driven and increasingly autonomous fault diagnosis. Through pre-training on large-scale general corpora and domain data, cross-task transfer, cross-modal representation learning, and reasoning capability can be acquired. In task-driven diagnosis, LLMs can be introduced through instruction tuning or in-context learning. Fault identification, operating-condition interpretation, and maintenance suggestion generation can then be performed within a single unified framework. Good generalization under operating-condition variation has also been reported [68]. LLM can systematically decompose user tasks and integrate expert knowledge to handle each subtask. This approach enhances LLM’s ability to effectively manage the entire task process [69]. Large language models such as GPT3.5, GPT4, and ChatGLM have demonstrated strong cognitive capabilities through perceptual learning and achieved notable results in multiple fields. These generative models provide users with responses that meet their expectations through cognitive capabilities supported by perceptual learning. For fault diagnosis, manual intervention can be reduced. Data-driven evidence and domain knowledge can be combined more tightly. In particular, retrieval-augmented generation (RAG), knowledge-graph retrieval and reasoning, and digital-twin simulation data can be integrated to enable deep collaborative diagnosis across “data–knowledge–physical–virtual models”. Several multimodal LLM-FDD frameworks have demonstrated that using GPT-like models combined with simulation-generated data and knowledge graphs can not only improve the accuracy of fault detection in complex systems but also maintain high robustness in scenarios with imbalanced samples and few-shot samples [70]. Overall, large language models provide a unified and scalable intelligent infrastructure for fault diagnosis, and their collaboration with digital twins and knowledge graphs is generally recognized as a significant development direction for the next generation of highly reliable and interpretable fault diagnosis.

The fusion paradigm of “LLMs + DTs + KGs” technically integrates their complementary advantages to overcome individual limitations through closed-loop synergy across data, knowledge and model layers. Digital twins address LLMs’ reliance on large-scale labeled real fault data and KGs’ scarcity of high-quality fault corpora. They generate high-fidelity synthetic fault data via physical–virtual mapping and controlled fault injection to support LLMs’ pre-training/fine-tuning and KGs’ automated entity-relation extraction. Meanwhile, LLMs mitigate DTs’ high modeling costs and poor real-time adaptability through parameter-efficient optimization and cross-modal generalization. This generalization enables parsing engineering documents to assist DT model generation and dynamic adjustment of DT parameters using real-time sensor data. Knowledge graphs in turn resolve LLMs’ “black-box” interpretability and hallucination risks. They provide structured domain knowledge (e.g., fault causal chains, component topology) as logical constraints for LLM reasoning, which facilitates traceable fault propagation analysis and evidence-grounded diagnostic outputs. They also compensate for DTs’ insufficient mechanism modeling by encoding expert rules and physical laws to guide DT simulation accuracy. Additionally, LLMs enhance KGs’ scalability by automating fine-grained knowledge extraction from unstructured data (e.g., maintenance logs) and resolving semantic inconsistencies in multi-source KG fusion, thus forming a self-evolving ecosystem. In this ecosystem, digital twins supply controllable data for model and knowledge updates, knowledge graphs provide interpretable knowledge for constrained reasoning, and LLMs serve as the core hub for cross-modal integration. They dynamically optimize the entire diagnostic pipeline to simultaneously overcome LLMs’ data dependency and poor interpretability, DTs’ high cost and low adaptability, and KGs’ inefficient construction and slow evolution.

### 1.3. Structure and Main Contributions

The main contributions of this paper are as follows. First, traditional machine learning, digital twins, knowledge graphs, and large language models are integrated into a unified review framework for the first time. The collaborative relationships among these technologies are clarified. Second, these approaches are systematically analyzed from three perspectives: theoretical principles, application scenarios, and advantages and limitations. A comprehensive technical comparison is thereby provided. Third, a fusion diagnosis paradigm of “large model + digital twin + knowledge graph” is proposed. New directions are offered for addressing current bottlenecks in the field. The remainder of this paper is organized as follows. Section 2 reviews traditional machine learning methods. Section 3 describes applications of digital twins and knowledge graphs. Section 4 analyzes large-model-driven fault diagnosis techniques. Section 5 compares the technologies and discusses fusion modes. Section 6 summarizes key challenges and future research directions. Section 7 concludes the paper.

## 2. Applications of Traditional Machine Learning in Fault Diagnosis

### 2.1. Supervised Learning Methods

**Figure 2:**
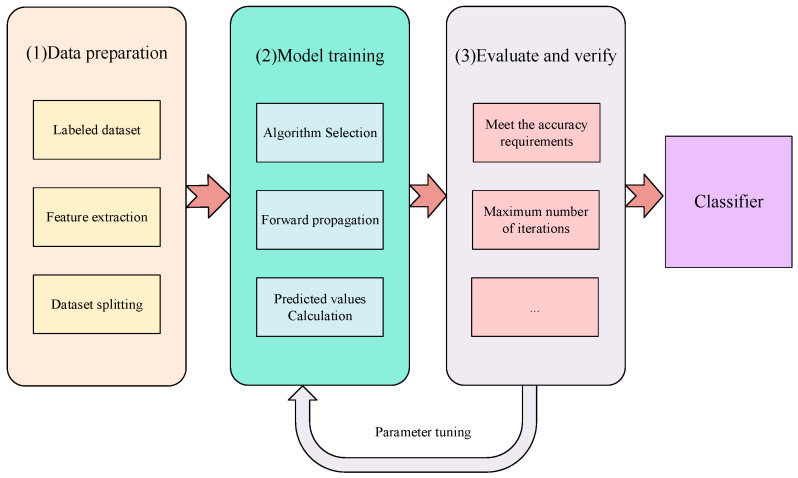
Schematic Diagram of the Supervised Learning Process [71].

**Figure 3:**
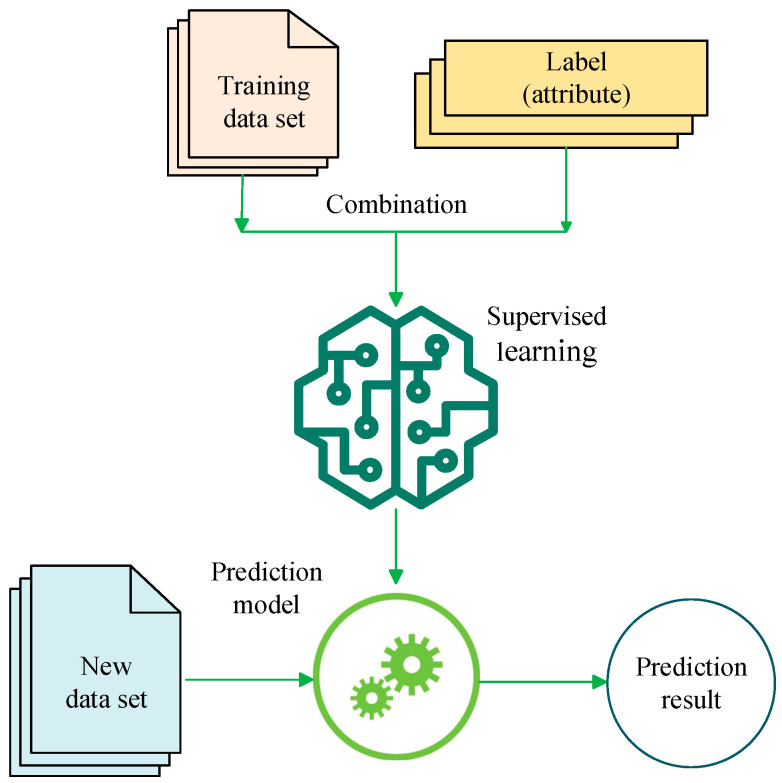
Supervised Learning-based Prediction Model Combining Data Set and Attributes.

Supervised learning is widely used in data-driven fault diagnosis. Historical samples with explicit health and fault labels are used for training, so as to allow for a classification or regression model to be fitted. The mapping between input features and fault categories is then learned, and identification and prediction of unknown states can then be achieved. The diagnostic workflow of supervised learning is illustrated in Figure 2. The main task of supervised learning involves the construction of an estimator that can predict the label of an object based on a given feature set. Model parameters are updated by comparing predicted outputs with ground-truth labels. Learning is thus realized through iterative error minimization [71]. Typical supervised learning algorithms include support vector machines (SVMs), random forests (RFs), gradient boosting decision trees (XGBoost), and traditional artificial neural networks (ANNs). As shown in Figure 3, this figure shows a supervised learning-based prediction model combining data set and attributes. The algorithm in this model distinguishes the observed data *X*, where *X* is the training data, which in most cases is structured data received by the model during the training process. In this process, the supervised learning algorithm constructs a predictive model. After the model training is completed, the fitted model will attempt to predict the most likely labels of the new sample set *X* in the test set [72].

Applications of traditional machine learning are listed in Table 2. SVMs are widely used in supervised fault diagnosis. High-dimensional feature spaces can be handled effectively, and strong generalization is often achieved. SVMs have therefore been reported to perform well in few-shot and high-dimensional fault diagnosis settings [73]. Good performance can be obtained with small training sets, which makes SVMs suitable for industrial scenarios where labeled samples are limited. SVMs can handle nonlinear problems by selecting appropriate kernel functions. Vibration feature analysis was used to diagnose defects in various components of rotating machinery bearings. Fisher score (FS) and genetic algorithm (GA) feature selection methods were integrated [74].

In addition, an SVM classifier with efficient hyperparameter tuning was then applied. An accuracy of over 99% was reported. Accurate classification of bearing defects was thereby achieved. Fault diagnosis was conducted on electric vehicle batteries based on multi-class support vector machines (MS-SVM) [75]. This method reduces the dependence on data volume while improving diagnostic accuracy and speed. The percentage of faulty turns in short-circuited windings was determined through new mathematical parameters proposed based on wavelet analysis. Classification was then performed using multi-class support vector machines. An accuracy of over 96% was reported. Strong capability of SVM-based methods for motor fault severity estimation was thereby indicated [76]. RF and XGBoost reduce the risk of overfitting through ensemble learning and improve robustness under complex operating conditions. A model of a marine diesel generator system was constructed, and an RF-based diagnostic model was then applied [77]. Hyperparameters were optimized using the IVY algorithm. Fault identification, diagnosis, and classification were performed within this framework. According to the reported results, the IVY-RF method distinguished normal and fault states with 100% accuracy. A grey wolf optimization (GWO)-based random forest (GWO-RF) algorithm was proposed [78]. The grey wolf optimization algorithm is used to optimize the total number of decision trees and the maximum depth of decision trees, effectively balancing the accuracy and generalization ability of the random forest model and significantly improving the accuracy of transformer fault identification. Decimation-in-time fast Fourier transform (DIT-FFT) was applied to rolling-bearing vibration signals, XGBoost was then trained on the transformed training set and evaluated on a validation set, and rapid fault-type identification was reported with this pipeline [79]; electromagnetic field simulation was used to obtain induction-motor stator currents, variational mode decomposition (VMD) was performed on the three-phase currents (A/B/C), approximate entropy was extracted as the fault feature, and high-precision motor fault diagnosis was obtained using XGBoost [80]; artificial neural networks (ANNs) enable nonlinear fault pattern recognition through multi-layer feature learning, and in motor-current-based induction-motor diagnosis, discrete wavelet transform (DWT) was first used for frequency-domain decomposition followed by ANN-based classification, where 100% accuracy and low test loss were reported when the tanh activation function was adopted [81]; strong cross-domain capability was also reported for an ANN-BiGRU domain adaptation framework in aircraft engine fault diagnosis, achieving an average accuracy of 92.6%, which supported the robustness and practical effectiveness of ANN-based methods in engine fault detection [82].

The main limitation of supervised learning in fault diagnosis is strong reliance on large volumes of high-quality labeled data. In industrial environments, real fault events are relatively infrequent, particularly severe faults and rare fault modes. Such samples are typically obtained only when equipment experiences actual failures, after which expert annotation is required. This process is time-consuming and costly. Safety risks and production interruptions can also be introduced. When fault samples are scarce, trained classifiers often fail to cover the full fault space, and recognition of rare or emerging faults is consequently weakened. As a result, deployment of conventional supervised models in real industrial sites is strongly constrained by label scarcity and label incompleteness [83]. Generalization is also limited. When equipment types, operating conditions, or environmental factors change, substantial performance degradation is often observed [84]. In addition, weak capability is typically exhibited in complex multi-coupling conditions and in the presence of previously unseen fault modes. Even when transfer learning is applied, severe misclassification can occur when novel fault categories appear in the target domain [85]. Finally, supervised learning is commonly implemented through offline batch training, and weak responsiveness to data dynamics is often reported. Real-time monitoring performance is therefore limited. Collectively, these issues restrict the practical adoption of supervised learning in complex, time-varying industrial environments [86].

### 2.2. Unsupervised Learning Methods

**Figure 4:**
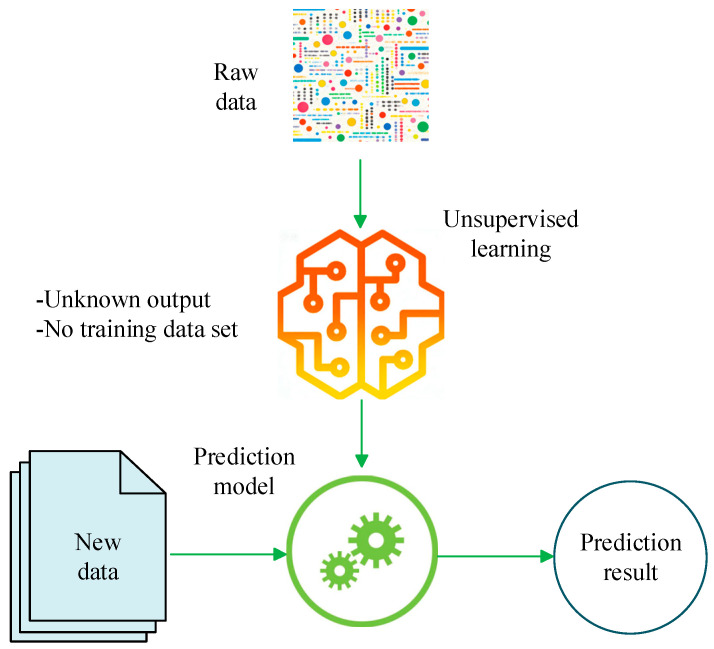
Unsupervised Learning-based Prediction Model for Fault Diagnosis.

Unsupervised learning is based on datasets without labels, realizing fault detection and clustering by mining the intrinsic structure of data [87]. Training can therefore be conducted using only normal-condition data, which are usually easier to acquire in sufficient quantities [88]. When unsupervised feature learning is effective, informative latent factors can be captured from raw measurements. Discriminative information can be amplified, and irrelevant variability can be suppressed [89,90]. Compared with supervised learning, manual labeling is not required, and high labeling cost and difficulty are avoided. Secondly, it can adapt to dynamic environments, that is, when the data distribution changes over time, the unsupervised model can capture new patterns in real-time through online learning. Typical algorithms include K-means, DBSCAN, autoencoder (AE), and generative adversarial network (GAN) [91]. As shown in Figure 4, this figure shows an unsupervised learning-based prediction model. In this type of model, sparse filtering is applied to extract local discriminative features from raw vibration signals. Signal-level representations are then obtained by averaging the learned local features. Then, SoftMax regression is used to classify health states based on these learned representations [92].

K-means performs fault clustering by minimizing within-cluster distances and is well suited to large-sample settings with reasonably separable fault modes. Computation is simple and convergence is typically fast, but degraded performance is often observed when clusters are non-convex or strongly overlapping. A deep architecture that combined transfer sparse autoencoders (SAE) with local maximum mean discrepancy (LMMD) was used to learn a shared latent feature space, after which K-means clustering was applied to fuse source- and target-domain information in that space; diagnostic accuracy was reported to exceed that of an MMD-based baseline [93]. In vehicle fault diagnosis, abnormal-data identification accuracy was improved by an enhanced K-means variant designed to mitigate limitations of standard K-means [94]. For rolling bearings, unsupervised feature analysis was used to study feature combinations, and accuracies above 99% were reported when simple time-domain and frequency-domain features were extracted and paired with common unsupervised diagnostic methods [95]. A UAV fault detection system was also developed based on related unsupervised learning ideas [96]. Experimental evidence has indicated that UAV in-flight faults can be detected effectively by combining vibration data with k-means clustering. DBSCAN, in contrast, performs density-based clustering and is well suited to non-convex fault clusters; it is also robust to noise and does not require the number of clusters to be specified in advance, which makes it appropriate when fault modes are unclear or measurements are heavily contaminated. DBSCAN has been applied to dissolved gas analysis (DGA) data for oil-immersed transformers, where density clustering was used to infer transformer fault types [97]. For battery systems, a PSO-SA-DBSCAN scheme was integrated into an online battery management system and a cloud monitoring platform, and electrochemical impedance spectroscopy and voltage measurements were used to diagnose abnormal degradation, thermal runaway, and sampling faults, with higher accuracy and lower false-alarm rates than conventional approaches [98]. Autoencoders learn nominal data structure through an encoder–decoder reconstruction process and can extract compact latent representations; anomalies can be flagged when inputs deviate from the learned normal distribution, which supports adaptation to diverse fault types. A high-precision LFDD method was developed by combining a gated recurrent unit autoencoder (GRU-AE) with random forests for fault detection in the control rod drive mechanism of pressurized water reactors, and high accuracy was reported on imbalanced real datasets [99]. Wavelet packet decomposition has also been combined with AE to decompose engine noise and noisy fault signals into frequency bands, after which band-specific datasets were used for training and evaluation; improved denoising performance was reported across multiple signal-to-noise ratios relative to alternative methods [100]. Generative adversarial networks address sample scarcity by generating synthetic fault samples that approximate real data, and they are particularly valuable under class imbalance, where training sets can be augmented to improve robustness and accuracy. A separation classifier was integrated into a GAN framework to generate multi-modal fault samples, and a Wasserstein loss with gradient penalty was introduced to improve distribution matching between generated and real fault samples; high accuracy was reported on two bearing datasets [101]. An improved GAN training strategy was further proposed by incorporating gradient normalization constraints and hinge loss to stabilize adversarial learning and enhance discriminative capability in unlabeled data; the resulting discriminator was fine-tuned with a small-labeled set and then used for fault identification, with effectiveness demonstrated on two induction-motor cases [102]. Overall, unsupervised methods have shown clear advantages in fault diagnosis, particularly when fault modes are unknown and labeled samples are scarce, and combinations of k-means, DBSCAN, autoencoders, and GANs have been reported to improve diagnostic accuracy and timeliness while supporting earlier warning for industrial equipment.

Although unsupervised learning provides an effective path for fault diagnosis without fault labels and the ability to discover unknown fault modes, it also faces significant limitations in practical industrial applications. First, compared with supervised learning, its fault classification accuracy is usually lower. This makes it difficult to achieve precise positioning of fault types. For example, when systematically evaluating various unsupervised anomaly detection algorithms, unsupervised learning methods have unstable classification and recognition performance under high-dimensional complex industrial data or mixed multiple fault modes [103]. Secondly, unsupervised methods are considerably sensitive to noise interference and data distribution drift. In real industrial environments, sensor data is often accompanied by environmental noise and changes in operating conditions, and the diagnostic performance of unsupervised models will decrease significantly [104]. Additionally, interpretability of unsupervised clustering results is often limited. Even when an “abnormal” state is detected, root-cause analysis is typically difficult. Clear attribution to a specific component, subsystem, or physical mechanism cannot usually be provided [105]. Therefore, although unsupervised learning is attractive when samples are scarce or fault categories are unknown, several bottlenecks remain. Diagnostic accuracy can be limited. Robustness to industrial noise and operating-condition drift is often insufficient. Interpretability is typically weak. Engineering stability in long-term deployment can also be difficult to guarantee. These limitations have prevented unsupervised methods from broadly replacing supervised learning in industrial-grade fault diagnosis.

## 3. Fault Diagnosis Driven by Digital Twins and Knowledge Graphs

### 3.1. Applications of Digital Twins in Fault Diagnosis

#### 3.1.1. Technical Principles and Architecture

Digital twins (DTs) integrate physical entities, virtual models, and twin data to simulate real-world objects or systems. Operational behavior of the physical system can be mapped into the virtual space. Simulation and state prediction can then be performed under predefined conditions. DTs can therefore be viewed as a high-fidelity mirror system that supports refined functions such as control, predictive optimization, simulation, and system evaluation [106,107]. DTs consist of three main elements: physical entity, virtual model, and information connection [108]. DTs construct a physical–virtual mapping system through three elements: physical entity, virtual model, and data link, realizing full-life cycle perception, simulation, and fault prediction of equipment.

**Figure 5:**
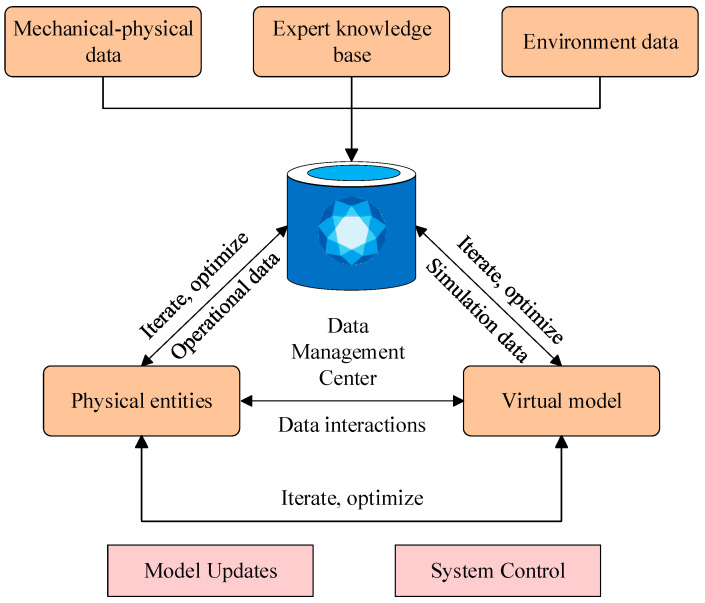
DT Five-Dimensional Model [114].

The concept of digital twins has been continuously improved with the continuous development of the Internet of Things [109], cloud computing [110], artificial intelligence [111], virtual reality [112], and other advanced technologies [113]. A five-dimensional digital twin model was extended from the earlier three-dimensional formulation by Tao et al. [114]. Physical, virtual, connectivity, data, and service dimensions were included, as illustrated in Figure 5.

High-fidelity virtual mapping of physical entities is regarded as the foundational capability of digital twin technology. This mapping is typically supported by four elements: twin data, the virtual mirror, information interaction, and the physical entity [115]. This is the foundation for realizing the Industrial Internet and intelligent operation and maintenance of equipment. DT technology can be used to monitor the actual behavior of objects or systems, but it usually requires a large amount of data and computing power to support model construction and operation, which is used to describe the operating mechanism of objects or systems in the real world. These data can be obtained through sensors or other methods and used to train models. Under predefined conditions, system behavior can then be predicted with high accuracy. Problems can be detected and addressed in a timely manner, which supports fault diagnosis and predictive maintenance of critical equipment components. For digital-twin-enabled intelligent fault diagnosis, the central requirement is the construction of a high-fidelity virtual mirror for core components or subsystems. Representative applications include degradation anomaly detection for wind turbine bearings [116], fault diagnosis for aero-engine blades [117], and operation-and-maintenance visualization for factory equipment [118].

**Figure 6:**
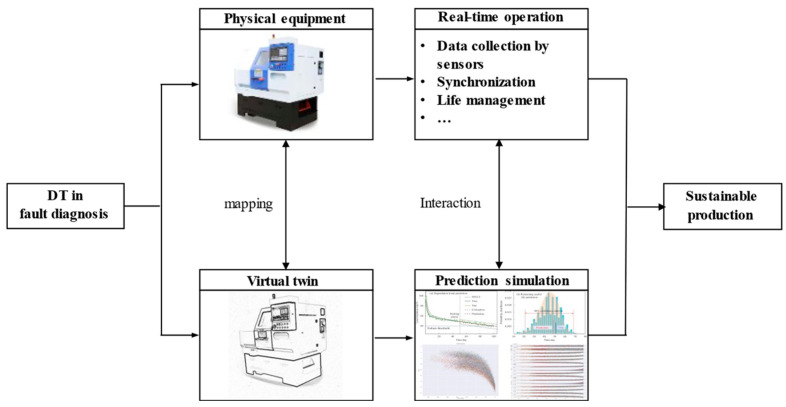
Technical Framework of DT-Driven Fault Diagnosis.

The technical framework of DT-driven fault diagnosis is shown in Figure 6. Digital twins generate synthetic fault data through a rigorous technical process centered on high-fidelity physical–virtual mapping by first constructing a multi-physics coupling virtual model based on the physical entity’s structural parameters material properties and operating mechanisms. This model integrates mechanical electrical and thermal dynamics to replicate real-world behavior followed by real-time data synchronization achieved via sensor networks IoT devices and data assimilation techniques. These techniques fuse physical operational data such as vibration and temperature with virtual model parameters to maintain temporal-spatial consistency. The process then involves controlled fault injection in the virtual space which includes parameter perturbations such as adjusting component stiffness for wear faults mechanism-based fault modeling such as simulating gear tooth breakage via structural deformation and degradation trajectory reproduction. This injection generates diverse fault scenarios including rare faults and multi-fault coupling that are difficult to obtain physically. Ultimately the virtual model runs iterative simulations under variable operating conditions such as load and speed to output large-scale synthetic data covering fault initiation propagation and deterioration. For scientific validation the synthetic data undergoes three key steps consisting of statistical consistency verification physical mechanism alignment and diagnostic performance validation. Physical mechanism alignment ensures synthetic fault evolution adheres to known mechanical and electrical principles such as matching vibration harmonic changes with theoretical fault modes. Diagnostic performance validation includes training fault diagnosis models on synthetic data and then testing on real-world datasets to evaluate generalization. This evaluation involves verifying accuracy recall and robustness across cross-device and cross-condition scenarios. It also entails iteratively refining the virtual model based on validation residuals to minimize physical–virtual discrepancy. This process ensures synthetic data is both physically meaningful and diagnostically effective for model training.

#### 3.1.2. Application Scenarios and Advantages

By integrating real-time monitoring, predictive analysis, and personalized maintenance insights, DT technology provides an advanced platform for industrial fault diagnosis (IFD). Synergy between physical assets and their digital counterparts can improve diagnostic accuracy and adaptability beyond conventional approaches. A basis is thereby established for more reliable, efficient, and responsive fault diagnosis in industrial environments [9]. In recent years, as DT technology has gained importance, its use in fault diagnosis has continued to expand. As shown in Table 3, representative applications of DTs are listed in this table.

In industrial manufacturing, DT-enabled intelligent fault diagnosis has been increasingly adopted as a high-impact approach. A high-fidelity digital twin, synchronized with real-time operational data, can be used to monitor equipment states continuously and to forecast degradation trends. Diagnostic efficiency and accuracy can therefore be improved, potential failures can be anticipated, downtime can be reduced, and maintenance planning can be optimized. Bearing-oriented digital twins have been implemented using CAD and simulation platforms, and accurate fault detection and degradation assessment have been achieved when multiple intelligent algorithms were integrated; simulated fault data and transfer learning were also used to mitigate limited fault data and constrained computing resources [119]. Online bearing fault detection has been realized by constructing a virtual model from an established dynamic model and updating it via online learning, while simulated numerical datasets were used for machine-learning-based classification; real-time fault probability estimation and parameter-adjustment feedback were enabled [120]. For gearbox unbalance diagnosis, nonlinear dynamics were analyzed and a twin-based dynamic simulation model was used to generate high-fidelity fault data, which reduced dependence on real fault acquisition and improved fault discriminability [121]. Physical–virtual data fusion has been applied to automotive gearbox diagnosis, and effectiveness was validated under multiple operating conditions [122]. In production-line settings, an improved random forest model was trained on DT-simulated balanced datasets and transferred to an automotive rear axle assembly line via transfer learning, achieving 97.8% accuracy [123]. Simulation-to-physical bearing diagnosis has also been supported by enhanced meta-transfer learning, with an average accuracy of 95.18% reported [124]. DT-based diagnosis of wind-turbine planetary gears has been implemented using empirical mode decomposition and an atom-search-optimized SVM, while model parameters were refined iteratively using diagnostic feedback [125]. For induction motors, a multi-physics DT constructed in COMSOL 6.0 was used to transfer virtual-space data to sparsely labeled physical datasets, and diagnostic capability was enhanced accordingly [126]. In the energy domain, DT-based fault diagnosis has been extended to diverse assets and has improved system intelligence and operational efficiency. For proton exchange membrane fuel cell systems, fault data were generated through model-level fault injection in high-order models and were combined with deep learning to diagnose multiple subsystem faults without relying on real labels [127]. Fault detection has been implemented for urban distributed photovoltaic systems using DT-based frameworks [128]. For chiller units, a DT mapping model was coupled with a stacked sparse autoencoder to support real-time defect diagnosis and result verification, and diagnostic accuracies above 90% were reported across fault severities [129]. For gas-path health management, DT-based early warning has been applied to measured LM2500+ data, and improved accuracy and efficiency were reported [130]. Battery energy storage DTs have been designed to provide updated behavior predictions, supporting condition monitoring, fault detection, battery management, and cyberattack detection and mitigation [131]. In nuclear applications, Bayesian reasoning has been integrated with DTs to infer fault probability distributions directly from real-time sensor streams [132]. For transformer winding faults, DTs have been combined with vibration analysis, wavelet-based feature extraction, and probabilistic neural networks to enable efficient and accurate detection [133]. In aerospace, DT-based intelligent diagnosis has been positioned as a key enabler for life-cycle support across design, manufacturing, operation, and maintenance. A DT of a hypersonic vehicle was constructed to simulate multiple fault states, and accurate identification was enabled through multi-scale feature extraction, residual self-attention feature enhancement, and GRU-based feature fusion [134]. Component-level mechanism models have been fused with data-driven models (e.g., PSO-XGBoost) using low-rank multimodal fusion, and an SSAE-based engine DT framework was formed for performance diagnosis; low parameter-prediction error and high gas-path fault diagnosis accuracy were reported [135]. Remaining useful life estimation has been supported by DTs built from sensor and IIoT data, with LSTM-based updates applied to track degradation and refresh RUL estimates [136]. Real-time DT monitoring platforms have been developed to provide interactive services based on multi-source, multi-dimensional data and complex analytics, with feasibility verified in large aircraft component assembly scenarios [137]. Deep multimodal fusion has also been introduced to integrate physical and simulation data within aero-engine DT frameworks, enabling real-time monitoring and high-precision fault detection [138].

The verification of accuracy and credibility of DT requires a closed-loop process that encompasses physical–virtual consistency assessment model drift detection and long-term maintenance. Physical–virtual consistency assessment proceeds from three aspects. It first focuses on the data layer through statistical tests including mean deviation and correlation analysis to verify the distribution consistency between virtual and real sensor data. It then targets the physical mechanism layer to ensure alignment between the multi-physics field coupling laws of the virtual model and actual equipment with vibration frequency and temperature conduction adhering to dynamic principles. It finally covers the diagnostic performance layer where diagnostic models trained with virtual generated data are tested using real fault data. Such tests demand an accuracy deviation of no more than 3 percent. Model drift detection involves real-time monitoring of characteristic differences between virtual outputs and physical feedback. Examples of such differences include a vibration peak deviation exceeding 5 percent and an increase in fault diagnosis delay of no less than 20 ms. This monitoring triggers drift alerts by considering equipment operation duration and working condition changes such as load fluctuations and sudden changes in ambient temperature and humidity. Long-term maintenance adopts an incremental training strategy which integrates equipment aging data and new fault modes to continuously optimize virtual model parameters. It reduces cross-platform update costs through lightweight modeling and establishes a closed-loop mechanism of physical feedback parameter calibration and virtual iteration. This mechanism ensures the stability and reliability of the DT model throughout the entire equipment lifecycle. It also serves as a core prerequisite for the DT transition from laboratory verification to practical industrial deployment.

Thus, intelligent DT-driven fault diagnosis has many advantages that are not readily matched by traditional methods. First, physical–virtual interaction can promote controllable fault simulation and rapid verification, significantly reducing the cost and risk of physical experiments. Relying on the virtual environment, various extreme operating conditions and rare fault scenarios can be constructed without affecting the operation of real equipment for algorithm training and strategy verification. This solves the problems of difficult fault reproduction and high test costs in actual systems. Secondly, full-life cycle data integration supports the improvement of diagnostic comprehensiveness. Digital twins can continuously accumulate and update data throughout the entire cycle from structural design, manufacturing, assembly, and operation to maintenance. Through multi-source data fusion, fault diagnosis is shifted from reliance on local signals to a global understanding of system health. Identification of progressive degradation and cross-cycle evolution mechanisms can therefore be improved. In addition, diagnostic robustness is enhanced by tight coupling between high-fidelity physical models and data-driven models. Complementarity between mechanism awareness and data learning can be exploited. Diagnostic stability can thus be maintained under sensor anomalies, missing data, and operating-condition variation. Real-time synchronization and visualization further improve decision efficiency. Key-component states can be mapped continuously. Interpretable and traceable fault evolution trajectories can be presented to maintenance personnel. Diagnostic transparency and decision reliability are thereby strengthened. Overall, through physical–virtual fusion, mechanism constraints, and full life-cycle health management, digital twins can improve the accuracy, reliability, and applicability of intelligent fault diagnosis. A major development direction is therefore provided for future industrial health management systems.

#### 3.1.3. Existing Challenges in the Application of DTs in Fault Diagnosis

Although DTs provide a high-fidelity simulation and physical–virtual fusion paradigm for fault diagnosis of complex equipment, major challenges remain in engineering deployment. A primary issue is data acquisition and data quality. In DT applications, the accuracy, completeness, and reliability of collected data must be ensured. High-quality, trustworthy, and real-time data are essential, because DT model calibration, state synchronization, and downstream diagnostic performance are directly constrained by the data stream. Nonetheless, in practice, data acquisition can be impeded by sensor coverage limitations, measurement inaccuracy, sensor failures, network instability, and harsh environmental conditions. Incomplete, noisy, or distorted data streams can therefore be produced. Even when data are collected successfully, quality can still be degraded when cleaning, preprocessing, and verification are insufficient. This creates a critical bottleneck for DT systems, because model calibration, synchronization, and diagnostic reliability are highly sensitive to input quality. As a result, the efficiency of DT model construction and long-term maintenance can be reduced, and the potential of DTs for industrial digitalization and complex-system decision support can be constrained [139]. Secondly, persistent challenges are encountered in DT technology with respect to model construction accuracy and real-time updating. It is fundamentally required that the virtual model be made to represent the instantaneous state of its physical counterpart, while being updated dynamically as the physical system evolves. High-precision DT models typically depend on sophisticated algorithms and large volumes of accurate, time-synchronous data; however, such data are frequently unavailable or unreliable in physical systems operating in complex or harsh environments. Combining data processing and mining technologies with mathematical and physical models to map the real-time state of the operating system is particularly challenging in complex electromechanical systems involving oil, fluids, and gases. While DT deployment for relatively simple components (e.g., bearings and gears) can often be implemented with limited difficulty, model complexity increases sharply for large-scale assemblies, which constitute the majority of industrial products and equipment. As a consequence, model construction and real-time updating are made more demanding, and technical as well as resource requirements are increased accordingly [140]. During long-term operation, additional degradation mechanisms are introduced by sustained wear, continuously varying operating conditions, process changes, and even structural modifications. Under these circumstances, “model drift” is commonly observed, such that ongoing parameter updating, structural reconfiguration, and algorithm retraining are required. High iteration costs and elevated operation-and-maintenance burdens are therefore incurred. This limitation is especially pronounced in systems with strongly fluctuating conditions, including motors and rotating machinery, where maintenance complexity must be reduced without compromising diagnostic accuracy, yet this balance remains difficult to achieve [141]. Finally, the main challenges of integrating highly complex DT technology also involve technical compatibility and integration costs. Digital twins must be integrated with existing information and physical systems, such as product lifecycle management (PLM), supervisory control and data acquisition (SCADA), sensors, Internet of Things (IoT) devices, and control systems. Because substantial heterogeneity exists across these systems and devices, a highly flexible and interoperable DT platform is required for seamless integration [142]. As such, urgent problems to be addressed for DT-based fault diagnosis include the control of computational and maintenance costs while preserving high-fidelity multi-physics coupling, the realization of high-reliability and low-latency physical–virtual synchronization within edge–cloud collaborative architectures, and the preservation of long-term effectiveness and transferability of DT diagnostic models under multi-source heterogeneous data and complex operating regimes.

### 3.2. Applications of Knowledge Graphs in Fault Diagnosis

#### 3.2.1. Technical Principles and Construction Process

A knowledge graph (KG) is a method of visualizing entities, their attributes, relationships, and interconnections, enabling computers to better understand and utilize data. It is suitable for the semi-structured semantic representation of data in various fields, such as data mining, information retrieval, and natural language processing. Key technologies used in knowledge graphs include entity recognition, relationship extraction [143,144], knowledge representation [145,146,147], and reasoning [148,149,150]. Knowledge graphs can be leveraged for fault diagnosis and maintenance decision support. In this paradigm, feature vectors representing mechanical fault signatures are compared against, and reasoned over with, entities, attributes, and relations encoded in the graph, such that fault root causes can be identified rapidly and suitable maintenance actions can be recommended. The construction of a knowledge graph must be undertaken with careful attention to domain-specific context, semantic schema design, and operational requirements, as these elements determine both representational fidelity and downstream usability. Methodological choices typically include top-down schema-driven development, bottom-up data-driven extraction, or hybrid workflows that integrate both strategies to balance conceptual coherence with empirical coverage [151]. As shown in Table 4, representative applications of KGs are listed in this table.

**Figure 7:**
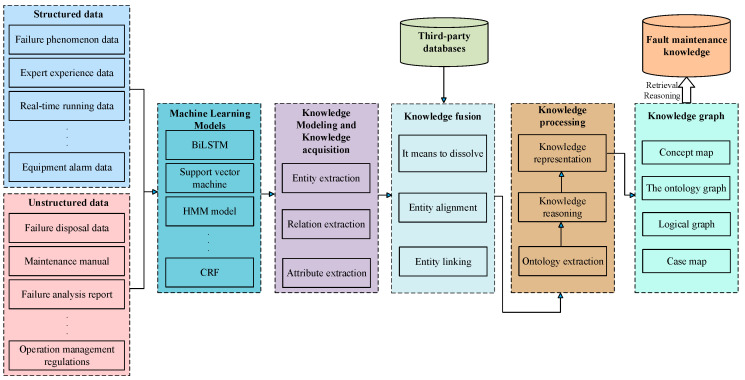
Knowledge Graph Construction Process [14].

**Figure 8:**
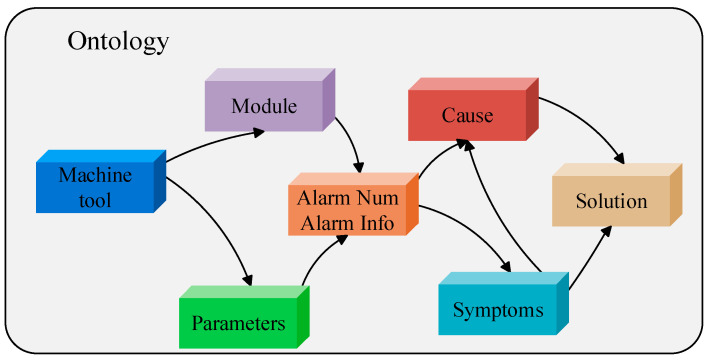
Ontology Design of Knowledge Graph [74].

As illustrated in Figure 7, a knowledge graph for intelligent production lines was constructed using a combined top-down and bottom-up approach. In addition to aligning with the construction principles of equipment-manufacturing fault knowledge graphs, this hybrid strategy is intended to preserve overall system integrity while improving flexibility for practical deployment and iterative expansion [1]. Consequently, knowledge-graph-based fault diagnosis has generally been reported to achieve higher accuracy and greater efficiency than conventional approaches [152]. Constructing an ontology for KG involves combining domain expert knowledge with data analysis using top-down and bottom-up methods. The top-down method uses expert systems and existing data patterns to guide the construction of knowledge graphs; while the bottom-up method identifies and integrates relevant knowledge from semi-structured or unstructured data through information extraction technology. These strategies effectively utilize in-depth domain knowledge and highlight the importance of data-driven methods in discovering and verifying new knowledge. For example, in the CNC system fault diagnosis knowledge graph project, the ontology design is based on general fault diagnosis standards and expert experience [153]. A canonical fault knowledge graph was constructed to represent core entities, including equipment components, fault types, fault features, and diagnostic methods. In the present study, seven principal entity classes were defined: equipment (machine tool), equipment module, parameter, alarm number/information, phenomenon (symptom), cause, and solution. The inter-entity relationships among these classes are summarized in Figure 8. An appropriate level of ontological complexity was maintained to support baseline fault-diagnosis functionality, while scalability was preserved to enable extension to broader application scenarios.

#### 3.2.2. Application Scenarios and Advantages

Knowledge graph technology can be regarded as an intelligent knowledge-base paradigm that combines artificial intelligence methods with conventional database technologies, and it is used to support the structured organization, storage, and retrieval of large-scale knowledge. In anomaly detection and fault diagnosis, knowledge graphs enable heterogeneous and fragmented industrial knowledge to be integrated, large volumes of domain text to be mined effectively, and latent diagnostic patterns or rules to be extracted and formalized [154]. In engineering practice, knowledge graph-driven fault diagnosis has been verified and applied in various complex equipment such as power systems, rotating machinery, and aerospace.

In the power industry, power operation and maintenance are of great practical significance for ensuring the safe and stable operation of power systems. A full life-cycle fault knowledge graph has been constructed for new power-system operational scenarios, where fault-type identification was achieved via graph reasoning and higher diagnostic efficiency was reported under complex series–parallel fault conditions [155]. To address the difficulty of applying conventional machine-learning methods to few-shot, high-dimensional transformer fault data, a risk prediction graph has been developed by incorporating operating conditions, temperature, and related variables; gradient boosting decision trees were then integrated to support fault-state evaluation and early warning [156]. Heterogeneous knowledge in the power-fault domain has also been structured using knowledge graphs to establish cross-knowledge associations, and an accuracy improvement of 1.79% was reported for a BiGRU-GA-based model [157]. In addition, a power-equipment defect knowledge graph has been established and a defect-record retrieval method based on graph search has been proposed. These have been shown to substantially improve retrieval performance for defect records [158]. An intelligent fault-diagnosis method for substation equipment has further been proposed by associating multi-modal information within a knowledge-graph framework [159]. Functional defect text data from secondary power-system equipment has been leveraged to build an intelligent diagnosis and auxiliary decision-making platform based on a BiLSTM-CRF model coupled with a knowledge graph, thereby supporting the diagnosis of secondary-equipment functional defects [160]. Moreover, entity recognition for the power-grid domain has been studied using transfer learning. The resulting entities have been used to support knowledge-graph-based power-grid fault disposal workflows [161]. In the aerospace domain, maintenance cost information and flight data have been combined to construct an aircraft fault knowledge graph, where fault phenomena, components, causes, and maintenance actions were mapped into a unified low-dimensional embedding space through relation extraction and representation learning; similarity-based retrieval was then used for intelligent auxiliary localization of faulty units, thereby reducing reliance on individual maintenance experience [162]. A fault-diagnosis knowledge graph has also been constructed for civil aircraft environmental control systems, together with a diagnosis-support algorithm, leading to improved fault localization accuracy and reduced maintenance costs [163]. Full life-cycle spacecraft information has been integrated using data-fusion techniques to construct a comprehensive knowledge graph; based on function–behavior–structure theory, a human–machine interface for spacecraft rolling-bearing fault diagnosis has been designed, and improved fault response capability has been demonstrated [164]. A knowledge graph for commercial aircraft faults has additionally been developed to enhance interpretability by incorporating fault logic within logical graphs; rapid localization of abnormal monitoring parameters and guided troubleshooting were reported based on existing information [165]. For aircraft control maintenance, a dedicated knowledge graph has been constructed and a hybrid information-extraction approach combining domain rules with machine-learning models has been proposed for entity recognition; evaluation on real airline manuals reported an average entity-recognition precision of 85% and an average relationship-extraction precision of 61% [166]. Further, a health-management knowledge graph construction method for aviation display equipment has been proposed using BiLSTM-CRF, enabling visualization of health-management-related fault information and providing support tools for aviation equipment health management [167]. Within intelligent manufacturing, a multi-level knowledge graph construction method has been proposed for novel rotating machinery equipment, where hierarchical modeling was performed at the structural layer, operating-condition layer, and fault-mechanism layer; fault diagnosis under missing features and operating-condition disturbances was then enabled via graph reasoning, thereby mitigating the degradation in accuracy typically observed in rule-based reasoning under incomplete information [168]. A knowledge-graph-based approach has also been introduced for fine-grained domain knowledge modeling and multi-source sensor data integration in machine-tool structural health monitoring; knowledge-graph querying and reasoning were used to support more intelligent and efficient sensor-data retrieval and analysis [169]. For CNC equipment, fault knowledge graph construction has been implemented using natural language processing, including BERT-based text classification and BiLSTM-based named entity recognition, to train, recognize, and model fault-corpus data for improved utilization of fault knowledge [170]. Finally, knowledge graphs have been applied to support lighting-system fault analysis by exploiting their associative reasoning and large-scale data analysis capabilities, thereby improving the reliability and intelligence of lighting-system fault diagnosis [171].

Fault diagnosis is inherently temporally evolving, but temporal modelling in knowledge graphs has only been briefly addressed; thus, it is necessary to expand relevant discussions. Temporal knowledge graphs go beyond static representation of entities and relationships by incorporating time dimensions such as timestamping the validity period of relationships and the sequence of fault events to construct dynamic knowledge structures that capture the time-varying characteristics of fault-related information. For fault propagation over time, it is essential to combine the temporal features of equipment operation to analyze the path of fault evolution from incipient stage to spread. Temporal knowledge graphs can effectively capture such dynamic propagation rules by clarifying the intensity of fault correlations and the scope of impact at different time nodes. Integration with time-series data requires aligning structured temporal knowledge with unstructured time-series data such as vibration and temperature sequences collected by sensors this integration enables complementary advantages between the two making structured knowledge guide the abnormal location in time-series data while time-series data verify the rationality of fault propagation paths in the knowledge graph ultimately improving the timeliness and accuracy of fault diagnosis.

Overall, knowledge graph technology has shown significant cross-industry and cross-equipment type advantages in the field of anomaly detection and fault diagnosis. Interpretability can be improved substantially, because relationships among equipment components, fault phenomena, inducing causes, and their interactions are explicitly modeled. Fault-propagation pathways can therefore be traced and presented clearly, such that a transparent and defensible diagnostic rationale is provided for engineers and reliance on individual experience is reduced, particularly under complex and multi-stage failure scenarios. Knowledge reuse and sharing are also enabled naturally. Historical structural information, operating rules, and fault mechanisms can be formalized in a unified semantic representation and transferred to similar assets or newly deployed equipment with limited reengineering, thereby providing directly reusable knowledge support and markedly shortening the development cycle of diagnostic models for new systems. At the same time, with the expansion of graph scale and the evolution of reasoning algorithms, the system can continuously absorb multi-source heterogeneous information, enriching the industry knowledge base while improving the coverage and accuracy of reasoning. These advantages together make knowledge graphs an important infrastructure for cross-scenario and cross-equipment type intelligent diagnostic systems, providing strong knowledge-driven capabilities for the efficient and safe operation of complex engineering equipment.

#### 3.2.3. Existing Challenges in the Application of Knowledge Graphs in Fault Diagnosis

Knowledge graphs represent a further technological progression of artificial intelligence for anomaly detection and fault diagnosis, yet several challenges remain when they are deployed in engineering practice. In fault diagnosis applications, a primary constraint is imposed by knowledge acquisition. Critical knowledge, such as fault mechanisms, symptom descriptions, and maintenance strategies, is often embedded in unstructured sources, including operation logs, maintenance records, and multimodal data streams. At present, knowledge graph construction is still heavily dependent on manual effort. Because data sources are diverse, data collection and preprocessing are associated with substantial workload and cost. Moreover, existing knowledge acquisition techniques frequently exhibit limited extraction accuracy, strong dependence on predefined templates, and insufficient robustness to long documents and domain specific vocabulary when complex industrial corpora are processed, such that extensive manual annotation and sustained expert involvement are still required for the development of high-quality fault knowledge graphs [172]. Secondly, lagging knowledge updates can substantially constrain the long-term usability of knowledge graphs in engineering practice. Modern equipment and production lines are frequently subject to software upgrades, hardware upgrades, process adjustments, the introduction of new operating conditions, and the emergence of new fault modes across the life cycle. If knowledge graph development continues to be conducted in an offline manner based on one time modeling and periodic manual maintenance, timely representation of changes in equipment structure, operating environment, and diagnostic logic will be difficult to achieve, and the resulting knowledge base may gradually become inconsistent with on-site conditions [173]. Existing industrial knowledge graphs often lack a tightly coupled update mechanism with real-time monitoring data, operation and maintenance event streams, and design change records. This leads to a gradual disconnection between knowledge granularity and timeliness and real system behavior, which not only weakens the adaptability of knowledge graph-based fault diagnosis systems to new faults and complex operating conditions, but also brings risks of outdated knowledge, outdated reasoning conclusions, and even misleading decisions. Therefore, the construction of an industrial fault knowledge graph that supports continuous evolution, traceable version management, and automated cleaning has become a prominent challenge. Additional limitations are imposed by semantic conflicts during multisource knowledge fusion and by insufficient cross domain knowledge transfer. Fault diagnosis is informed by heterogeneous information sources, and differences in coding granularity, naming conventions, and implicit semantic assumptions are frequently observed. When a unified ontology and strict semantic constraints are not provided, the mapping of multisource data into a single knowledge graph is likely to introduce duplicated entities, conflicting relations, semantic ambiguity, and inconsistent reasoning results. At the same time, many existing construction pipelines are primarily data-driven rather than knowledge-driven during ontology and schema design, and the resulting ontology is often overfitted to a single data source, which further weakens cross domain sharing and transfer capability. Thus, from a fault diagnosis perspective, it remains challenging to achieve high quality fusion of multisource heterogeneous fault knowledge while preserving semantic consistency and logical self-consistency, and to improve knowledge transferability across equipment types, operating conditions, and enterprise environments through ontology alignment, domain adaptation, and transfer learning, which continues to hinder the large-scale deployment of knowledge graphs.

## 4. Large Model-Driven Fault Diagnosis Technology

### 4.1. Technical Characteristics and Adaptability of Large Language Models

**Figure 9:**
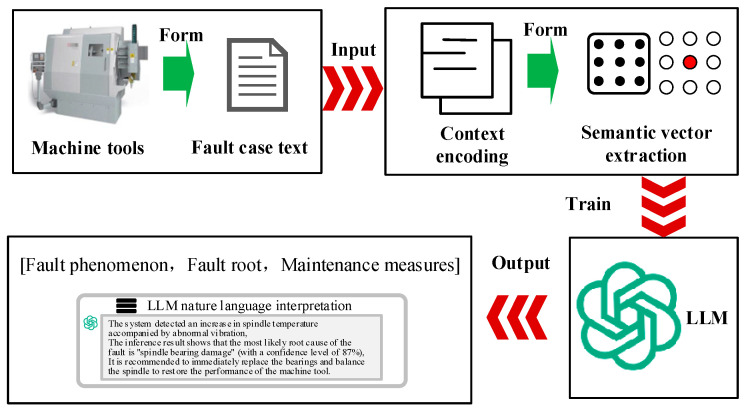
LLM-driven Fault Diagnosis Framework.

LLMs (such as GPT and GLM) are driven by scaling effects [174], involving the continuous increase in model size, training data volume, and computing resources used for training. LLMs have demonstrated capabilities such as in-context learning, instruction following, and step-by-step reasoning. Strong performance has been reported across a wide range of natural language processing (NLP) tasks [175]. Research on large language models has expanded from a single “language modality” to multimodal large language models capable of understanding “images”, “speech”, and other modalities [176]. Methods such as ViT, CLIP, and BLIP-2 have achieved cross-modal understanding between vision and text by aligning visual features with text features. In addition, Image-bind and Panda-GPT have enabled multimodal understanding and processing by mapping multiple modalities into a unified representation space. In fault diagnosis, large model technologies are increasingly being positioned as a key driver for improving diagnostic accuracy and efficiency, particularly within deep learning-based pipelines and large-scale data processing settings. As shown in Figure 9, this figure shows a multimodal large model framework for fault diagnosis. In this type of framework, data and text modalities are first aligned through training, and the large model is then fine-tuned for fault diagnosis tasks. At present, commonly adopted large model families include Transformer-based series models, pre-trained language models, and specialized fault pre-trained models designed specifically for fault diagnosis tasks.

When fault diagnosis tasks are processed, large language models exhibit distinct advantages. Firstly, strong capability for large scale data learning is provided. Training is typically performed on large and heterogeneous multisource datasets, and generalizable representations can be extracted from diverse inputs that include sensor measurements, equipment logs, fault reports, and operation manuals [177]. ViT-based models process image data by dividing it into multiple patches, thereby capturing long-range spatial dependencies in images [178]. In fault diagnosis, this technical characteristic enables the model to extract potential fault features of equipment from various types of data such as vibration signals and temperature changes. As such, efficient fault detection and prediction can be realized [179]. Secondly, strong context modeling capability is achieved, and long-range dependencies in time series signals can be captured. Because equipment state information is inherently time dependent, particularly under dynamically varying operating regimes, global temporal dependencies can be modeled by Transformer-based architectures through self-attention mechanisms, and weak or early fault indicators can be captured more effectively during equipment operation [180]. TimeSformer further strengthens this ability. By introducing a spatio-temporal attention mechanism and combining the temporal and spatial features of time-series signals, it can accurately model the equipment health optimization process [181]. Additionally, a pretraining and fine-tuning paradigm is typically adopted by large language models, such that adaptation to specific fault scenarios can be achieved with only a small number of labeled samples. Dependence on large, labeled datasets is therefore reduced substantially, which is particularly valuable in fault diagnosis where labeled samples are often scarce and expensive to obtain. The pretraining and fine-tuning strategy exemplified by BERT has been widely applied in natural language processing, and similar workflows have been extended to industrial fault diagnosis. By pretraining on equipment fault logs and maintenance records and then fine tuning for target assets or fault types, diagnostic efficiency and accuracy can be improved [182]. Finally, equipment faults are often the result of the combined action of multi-source data in actual industrial environments. This data includes multimodal data such as vibration, temperature, sound, and text. Transformer-based multimodal models can uniformly model data from different sources and perform cross-modal information fusion through sub-attention mechanisms. Through multimodal fusion, discrimination among diverse fault signatures is strengthened and robustness to complex and nonlinear fault modes is enhanced. This capability is particularly beneficial in multi sensor and multi equipment environments, where cross modal collaborative diagnosis can be supported more effectively.

### 4.2. Typical Applications and Innovation Directions

#### 4.2.1. Time-Series Fault Diagnosis

**Figure 10:**
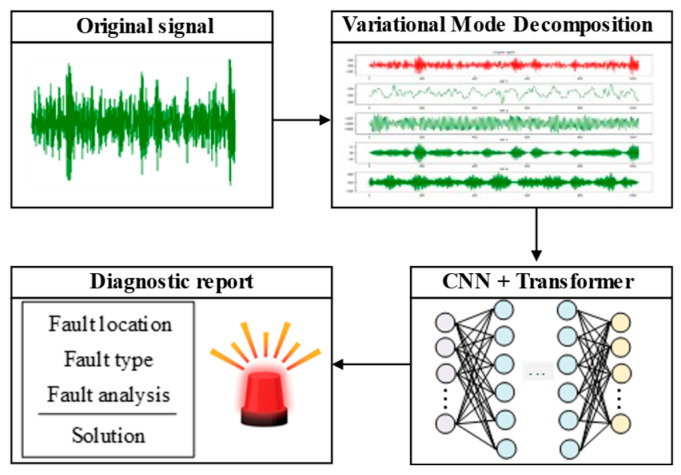
Transformer-Based Framework for Fault Diagnosis.

By leveraging self-attention mechanisms to model long range dependencies and cross channel correlations, Transformer architectures have progressively alleviated key limitations of conventional CNN and RNN approaches in time series fault diagnosis. In particular, constraints related to long sequence representation, sensitivity to variations in operating conditions, and restricted feature expressiveness have been mitigated. As shown in Figure 10, this figure shows a Transformer-based framework for fault diagnosis that can directly process 1D format data.

As shown in Table 5, representative applications of LLMs are listed in this table.

By designing a time series tokenizer to divide 1D vibration signals into several fixed-length subsequence tokens, and introducing a multi-head self-attention mechanism to model long-range correlations across cycles, end-to-end fault identification of rotating vibration signals is realized [183]. A Transformer-based motor fault diagnosis model has also been developed, where attention weights are adaptively allocated across modalities via multi head self-attention so that salient modalities and critical time intervals are explicitly emphasized [184]. A time series vision Transformer, referred to as TSViT, has been proposed by combining convolutional layers for local feature extraction from vibration signals with a Transformer encoder for long horizon pattern discovery in time series, and perfect accuracy has been reported on two test sets, indicating strong capability for rotating machinery fault diagnosis [185]. In addition, a time frequency Transformer model has been introduced for mechanical systems and signal processing, where a dedicated tokenizer and encoder module are designed to extract discriminative features from time frequency representations of vibration signals [186]. A hybrid architecture termed Dconformer has further been proposed by integrating multiscale convolutional feature extraction with Transformer self-attention, and denoising as well as multitask joint learning mechanisms are incorporated to strengthen representation of early and weak fault signatures under strong noise, resulting in improved diagnostic accuracy and noise robustness relative to pure CNN models on multiple datasets [187]. For deployment in constrained environments, a lightweight CNN Transformer framework has been presented and superior diagnostic performance has been reported on vibration datasets from planetary gearboxes and bearings, suggesting feasibility for resource limited scenarios [188]. A vision Transformer approach with frequency channel attention has also been proposed for intelligent rolling bearing fault identification, and strong cross condition diagnostic accuracy has been validated on two datasets [189]. Finally, a multiscale TransFusion model has been developed for rolling bearing diagnosis under multiple operating conditions, where a multiscale feature fusion module is used to extract low level information from time frequency signals and global dependency modeling by a Transformer is used to mine long cycle fault characteristics, enabling accurate bearing fault diagnosis across operating regimes with high robustness to noise contamination [190].

As inherently text-modal models, the integration of LLMs with industrial time-series data including vibration temperature and current as well as sensor signals hinges on modal unification and semantic alignment, with such deep integration achievable through three-tiered technical frameworks. The first framework is the linguistic encoding of industrial signals which converts unstructured industrial signals into semantic representations interpretable by LLMs thereby overcoming the limitations of direct numerical sequence processing. Time-series tokenization segments signals via sliding windows with vibration signals segmented using 50 ms windows as an illustration then extracts time–frequency features through wavelet packet decomposition and variational mode decomposition before encoding features of each window including mean variance peak value and approximate entropy into textual descriptions an example of such a description is a statement that the peak value of vibration signals in the 10–20 Hz frequency band attains 0.8 g with a variance of 0.03 exceeding the normal threshold by 20 percent. Signal-text prototype mapping pre-trains the corresponding relationships between signal features and textual descriptions a typical correlation is that inner race faults of bearings correspond to prominent 1x and 3x frequency components alongside dense time-domain peak pulses and constructs a cross-modal dictionary via contrastive learning enabling LLMs to reversely associate signal features through textual semantics. Multi-sensor signal fusion encoding leverages an attention mechanism to weight features of multi-source signals such as temperature pressure and current generating unified semantic text a representative instance is a record that the current distortion rate of phase A of the motor is 5 percent the bearing temperature reaches 78 °C and the 1x frequency peak value of vibration stands at 0.9 g which matches the fault characteristics of stator winding short circuits. The second framework is cross-modal alignment and knowledge enhancement which enhances the capability of LLMs to interpret industrial signals through technical strategies instead of relying merely on textual semantics. Dual-stage pre-training involves pre-training LLMs with extensive industrial logs and fault manuals in the first stage to establish semantic correlations among fault phenomena features and causes and introducing signal-text paired data such as vibration signals and corresponding fault descriptions in the second stage achieving modal alignment through contrastive learning so that LLMs can directly identify fault features from signal-encoded text. KG constrained alignment embeds industrial equipment topology and fault causal chains such as the sequence where gear wear induces increased 3x frequency of vibration and subsequently leads to reduced transmission efficiency into the reasoning process of LLMs and generates correlation prompts between signal features and fault entities through KG retrieval guiding LLMs to focus on critical signal indicators. The third framework is prompting engineering and lightweight fine-tuning which optimizes the adaptability of LLMs to industrial scenarios to balance performance and deployment costs. Fault diagnosis dedicated prompting templates are developed with structured prompting content incorporating known signal features the encoded text of signals equipment type such as wind turbine gearboxes and operating conditions such as rotational speed of 1500 rpm and load of 75 percent to guide the model to identify fault types locate fault components and formulate maintenance recommendations mitigating the hallucination issue of LLMs. Low-rank adaptation (LoRA) fine-tuning freezes the backbone parameters of LLMs and trains only the signal encoding layer and cross-modal attention layer to adapt to specific industrial scenarios such as wind power and rail transit the volume of fine-tuning data can be reduced to one-tenth of that required by traditional deep learning approaches with only 50 to 100 groups of labeled fault data deemed sufficient for effective adaptation.

#### 4.2.2. Few-Shot and Zero-Shot Fault Diagnosis

Large language models have significant advantages in representation learning, cross-task transfer, and parameter-efficient fine-tuning. As such, few-shot and zero-shot fault diagnosis are becoming critical directions of large model-driven intelligent maintenance. Firstly, large language models based on domain pre-training can learn general features on a large amount of unlabeled data, and can complete rapid adaptation in downstream tasks with only a small number of labeled samples. Secondly, by constructing a fault semantic space, knowledge graph, and attribute description, and combining with the representation ability of large language models, fault diagnosis under zero-shot conditions is finally realized.

**Figure 11:**
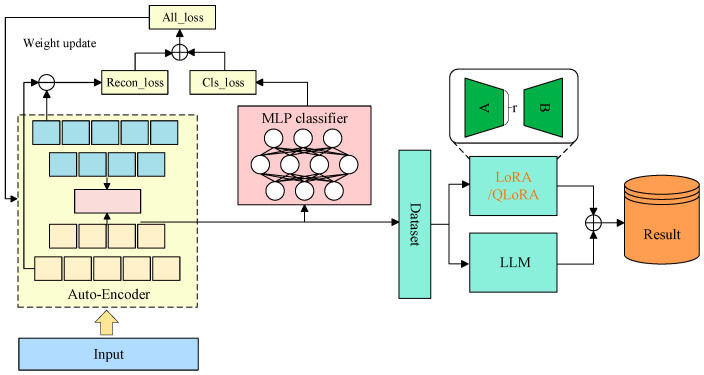
Fault Diagnosis Framework Based on Supervised Autoencoder [193].

For few shot learnings, a fault domain pretrained model termed EDformer has been developed for mechanical systems under limited sample settings. Self-supervised pretraining is first conducted on multisource signals using an encoding and decoding network, after which multisource information is fused via cross modal cross attention, and diagnostic fine tuning is performed with only a small number of labeled samples for downstream few shot tasks. Across eight diagnostic tasks on two multisource bearing datasets, an average classification accuracy of 98.94 percent has been reported [191]. Few shot diagnosis with noisy labels has also been investigated, and an enhanced Transformer with an asymmetric loss function has been proposed to enable diagnosis under different operating conditions when labeled data are both limited and imperfect [192]. In addition, a supervised autoencoder has been integrated with a large language model, as illustrated in Figure 11, where features extracted by the autoencoder are converted into text sequences and processed by the language model; with parameter efficient fine tuning, end to end fault classification is enabled and strong performance has been reported in feature extraction, diagnostic accuracy, and few shot adaptability [193]. A further approach has been proposed for autonomous underwater vehicle propeller vibration signals by combining time frequency and frequency domain information and expanding fault samples via a dual loss objective, after which the augmented samples and normal data are fed into a channel attention residual network that is fine-tuned with a large model, yielding an average accuracy of 96.81 percent [194]. A few shot methods based on the pretrained GPT 2 model has also been presented for full ceramic bearing fault diagnosis, motivated by the strong transfer learning and few shot capabilities of large language models that are pretrained on diverse text corpora and can therefore be adapted when labeled data are scarce [195]. For zero shot fault diagnosis, a framework that combines knowledge graphs and large language models has been proposed, where domain knowledge is embedded into the language model and diagnostic recommendations are generated through retrieval augmented generation, enabling identification without labeled samples in an aviation assembly setting. An additional large language model enabled zero shot framework has been developed by fine tuning a model with domain specific knowledge to capture information that is similar to manual annotations, and by using cross modal activation of relevant knowledge learned from domain documents to mitigate domain shift for previously unseen fault types; an average accuracy improvement of 9.83 percent for unseen faults has been reported [196]. Supervised fine tuning based on fault and non-fault labels has also been introduced to raise large language model diagnostic accuracy, with large gains reported for variable air volume box faults and chiller system faults, where average accuracy increased from 33.0 percent to 98.3 percent and from 36.0 percent to 99.1 percent, respectively [197]. For freight unmanned aerial vehicle fault diagnosis, a method that combines a multi-relational knowledge graph with a few shots chain reasoning strategy using a large language model has been proposed, where faster knowledge representation is attributed to the graph while diagnostic accuracy improvements are attributed to the reasoning component [198]. Finally, a Prototypical Vision Transformer has been developed for zero shot bearing fault diagnosis, where Vision Transformer feature representation is combined with prototype-based representation learning to improve performance under limited data availability [199].

#### 4.2.3. Multimodal Fault Diagnosis

Multimodal data fusion represents the core technology of modern fault diagnosis systems, which integrates heterogeneous data from various sources to provide a more comprehensive and accurate assessment of equipment conditions [200]. Fault diagnosis systems need to process various types of data in industrial environments. This data includes vibration signals, temperature data, pressure measurements, acoustic signals, image information, and text records. Traditional approaches have largely been based on feature level fusion and decision level fusion. Semantic correlations among modalities are often not modeled explicitly, and complementary meaning across modalities can therefore be underutilized. Large language models offer an alternative route for multimodal fusion. Data from heterogeneous modalities can be transformed into natural language representations, and fusion can be performed at the semantic level rather than only at the feature or decision level. The core concept is that natural language can be used as a universal interface language, through which heterogeneous observations are mapped into a unified semantic space. Semantic alignment represents a critical technology for multimodal fusion, as data from different modalities typically exhibit different feature spaces and representation methods, making it challenging to establish semantic correspondence between them. Compared to traditional fusion methods, LLMs can discover potential associations between different modalities by learning cross-modal shared representations [201].

**Figure 12:**
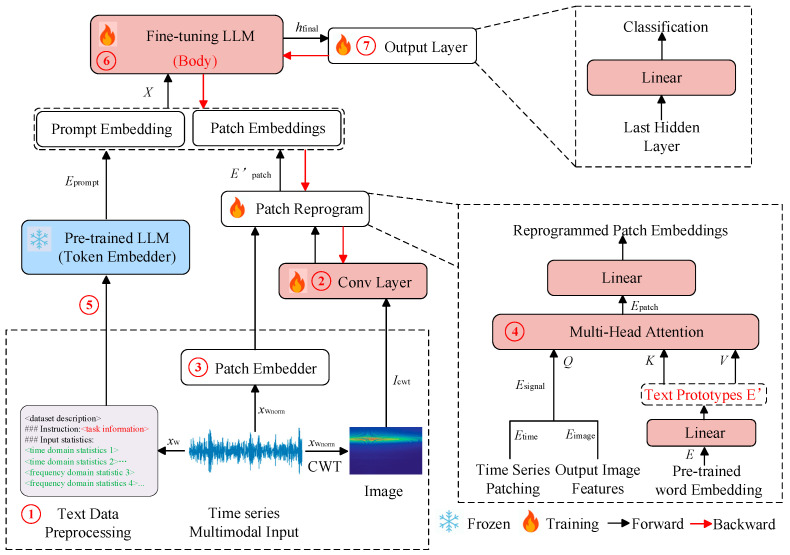
Fault diagnosis framework integrating pre-trained/fine-tuned large language models with patch reprogramming for multimodal (text, time-series vibration, image) data [206].

Multimodal fusion systems based on LLMs typically adopt an encoder–fuser–decoder architecture [202]. Modal encoders convert raw data into vector representations, multimodal fusers integrate cross-modal information, and decoders generate final diagnostic results and explanations. For numerical sensor data, encoders must transform time-series signals into natural language descriptions. This involves three steps: signal feature extraction, anomaly detection, and language generation. Modal alignment and learnable prompt fusion have been investigated for engineering time series data and background knowledge text. Alignment training in feature space was used to activate the large language model capability for interpreting engineering time series. A fuzzy semantic embedding strategy was introduced to alleviate pattern confusion, and learnable prompts were integrated with Low Rank Adaptation-based fine tuning to improve classification accuracy [203]. FaultGPT [204] fuses vibration time–frequency images with text instructions and labels to generate end-to-end diagnostic reports. For bearing fault diagnosis tasks, an accuracy rate of 98.7% is attained by FaultGPT through the fusion of vibration signals and textual instructions, which reflects a 4.3% performance enhancement compared with single-modal models. DiagLLM [205] combines envelope spectrum images with expert knowledge text to enhance interpretability. DiagLLM’s performance is assessed by its cross-device and cross-condition generalization capability, which measures the model’s stability when applied to diverse equipment and complex operating environments, as well as the quality of automatically generated fault reports, which evaluates the readability and completeness of diagnostic conclusions output by the large language model. It constructed “diagnostic visual instruction following” data, explicitly aligned feature descriptions with signal characteristics, and incorporated Parameter-Efficient Fine-Tuning (PEFT) to achieve physics-constrained reasoning. A multimodal fault diagnosis framework has been developed in which original vibration time series, two categories of time frequency images, and indicator text from a bearing system are jointly used to drive a large language model, as illustrated in Figure 12 [206]. In this framework, reprogrammed time series features and image features are converted into patch embeddings via text prototypes, and salient time domain and frequency domain indicators are injected into prompts to form prompt embeddings. In Data Encoding Module, the core function is to convert heterogeneous multimodal raw data into unified, model-recognizable vector representations, laying the foundation for cross-modal fusion. In Prompt Embedding Module, this module serves as a “bridge” between domain knowledge and the large language model, guiding the model to focus on fault-related key information and enhancing the interpretability and targeting of diagnosis. In LoRA Fine-Tuning Module, Low-Rank Adaptation fine-tuning is a parameter-efficient model adaptation technology, whose core function is to adapt the pre-trained large language model to the specific task of multimodal fault diagnosis without excessive computational overhead. The core of integrating LLMs with industrial signals lies in “signal semanticization + cross-modal alignment + industrial scenario adaptation,” which breaks the modal barrier through three layers of technologies: encoding, alignment, and fine-tuning. The FD-MVLLM architecture addresses the three major industrial pain points of safety, latency, and reliability through multimodal fusion, knowledge constraints, and lightweight deployment, thus providing an implementable solution for the engineering application of LLMs. In the future, it is necessary to further optimize the efficiency of signal encoding, the few-shot learning capability of LLMs, and the real-time data synchronization capability with Digital Twin (DT) to achieve the closed-loop diagnosis of “data–knowledge–model.”

A multimodal Transformer model has also been constructed by fusing vibration signals, temperature measurements, and related information to perform rotating machinery fault diagnosis, with markedly improved accuracy across multiple operating conditions relative to unimodal approaches. In addition, a fault detection and diagnosis framework has been proposed based on a multimodal large language model that fuses real measurements with virtual data generated by digital twins through cross modal attention, thereby maintaining strong diagnostic performance in industrial environments characterized by limited samples, numerous fault types, and complex operating conditions [207]. Finally, a fusion attention network based on multisource heterogeneous data has been developed by converting modality specific signals into images or feature representations and then applying multi head attention for fusion, which has been shown to improve both accuracy and robustness for bearing fault diagnosis [208].

### 4.3. Challenges in Large Model-Driven Fault Diagnosis

LLMs can support end to end fault diagnosis workflows, spanning report understanding, multisource data fusion, pattern recognition, root cause analysis, maintenance planning, and human–machine collaboration. Accuracy can be improved. Traceability can be strengthened. Decision support can be enhanced across multiple industrial sectors [201]. Nevertheless, it is essential to fully consider potential risks and numerous technical challenges when advancing LLM applications in fault diagnosis.

High computational and storage costs, together with difficult edge deployment, remains a primary constraint. Large language models are typically characterized by considerably large parameter counts and long inference pipelines. Significant overhead is incurred by attention computation and autoregressive decoding, and strict requirements for real time response, energy efficiency, and operational reliability are therefore difficult to satisfy on industrial edge devices. Additionally, industrial environments are often characterized by heterogeneous equipment and fragmented computing resources. As a result, engineering complexity in deployment and lifecycle management is further increased.

Limited interpretability and poor traceability of fault decision processes also remain significant constraints. Although quantification and attribution methods have been proposed, real time diagnostic requirements are often not satisfied in practice, particularly when explanations must be generated under strict latency constraints [209]. For example, Transformer-based large language models excel in time-series fault diagnosis, but their inference latency and memory consumption far exceed traditional models, severely limiting edge device deployment [210]. Further, the lack of transparency in large model reasoning processes is not conducive to fault tracing. In critical industrial domains, prediction interpretability represents a necessary condition, yet existing explanation methods remain inadequate for cross-modal diagnosis. The generative capabilities of LLMs may also introduce “hallucination” risks. When supporting evidence is limited, incorrect reasoning chains and unreliable diagnostic conclusions may be produced, and engineering safety can therefore be jeopardized [211]. Finally, weaknesses persist in engineering deployability and lifecycle management of intelligent diagnostic systems. Industrial environments are complex. High stability is required. Low latency is expected. Fault tolerance must be ensured. Meanwhile, equipment states and operating regimes evolve over time, and continuous model updating is needed to prevent performance degradation. As a result, the lack of standardized evaluation protocols, robust version management mechanisms, and practical online learning frameworks remains a major bottleneck that constrains the large-scale deployment of intelligent diagnosis solutions [212].

## 5. Challenges and Future Works

### 5.1. Core Challenges

With the continuous advancement of traditional machine learning, digital twins, knowledge graphs, and large language models in industrial fault diagnosis, the intelligent diagnosis paradigm is evolving toward an integrated intelligent system characterized by physical–digital fusion, knowledge-driven decision making, and large model reasoning. Nevertheless, numerous critical challenges remain in the practical application scenarios of industrial production.

First of all, the problems of insufficient data quality and imbalanced data distribution are prominent. Industrial field data are usually featured with strong noise interference scarce labeled samples and variable working conditions which hinder the extraction of stable discriminative features for diagnostic models. In addition, significant differences exist in the data distribution across various industries equipment types and working conditions leading to distinct domain shifts between the training datasets and practical application scenarios. This discrepancy greatly impairs the cross-scenario generalization capability of diagnostic models. Consequently, even with advanced architectures such as deep learning and Transformer models still struggle to adapt to complex working conditions including load variation rotational speed fluctuation and equipment aging [213,214]. Meanwhile fault samples are extremely scarce in industrial scenarios accompanied by severe class imbalance issues. Such issues easily induce overfitting of complex diagnostic models and further undermine their generalization stability [215].

Secondly the high construction and maintenance costs of digital twins and knowledge graphs have greatly restricted their large-scale application in industrial systems. The establishment of digital twins requires high-precision physical modeling real-time data synchronization and cross-platform system integration. It is difficult to fully model the multi-field coupling characteristics of complex equipment which often results in accuracy degradation or model drift in practical applications [216]. The construction of knowledge graphs is confronted with the difficulty of professional knowledge extraction the challenge of cross-domain knowledge fusion and the limitation of delayed knowledge updating. Automated knowledge extraction depends on high-quality industrial corpora. Existing methods achieve an accuracy rate of merely 60–70% in nested entity extraction, which fails to meet engineering requirements. This situation renders the constructed knowledge graphs hard to expand and update [217]. Meanwhile knowledge from different sources is prone to generate logical contradictions during the fusion process which causes unstable reasoning outcomes and thus makes it difficult to support continuous diagnostic tasks in the context of equipment technological iteration [218].

Thirdly, the practical deployment of large language models in industrial fault diagnosis is constrained by computational resource shortages, poor interpretability, and potential security risks. Large language models contain massive parameter scales which make their deployment on edge devices extremely difficult. Even with the adoption of model compression and quantization technologies it remains a great challenge to meet the real-time response requirements of industrial fault diagnosis [219]. For instance, Transformer-based large language models exhibit excellent performance in temporal fault diagnosis yet their high inference latency and excessive video memory consumption are far beyond those of traditional models imposing severe restrictions on their deployment on edge devices [220]. In addition, the black-box reasoning process of large language models is not conducive to fault root cause tracing. Predictive interpretability is an indispensable prerequisite in critical industrial fields whereas the existing interpretation methods are still inadequate for cross-modal fault diagnosis tasks. The generative capability of large language models also brings the risk of hallucinations where erroneous reasoning or unreliable diagnostic conclusions may be generated in the absence of sufficient evidence thus threatening engineering operational safety [221]. Finally, the intelligent diagnosis systems are still deficient in terms of engineering ability and full lifecycle management. The complex industrial field environment imposes stringent requirements on the operational stability inference latency and fault tolerance of diagnostic models. Meanwhile, the operating states of industrial equipment evolve over time, which necessitates continuous model updating to avoid performance degradation. The lack of standardized model evaluation systems unified version management mechanisms and efficient online learning frameworks has become a critical bottleneck that restricts the large-scale practical application of intelligent fault diagnosis technologies [222].

### 5.2. Future Development Directions

To address the described challenges outlined above, intelligent fault diagnosis is expected to evolve toward deep integration of knowledge, data, and models. Synergistic coupling of large language models, digital twins, and knowledge graphs is anticipated to become a primary driver of industrial transformation, enabling diagnostic systems that are more reliable, more generalizable, and more engineering ready.

First, data-level capability enhancement and cross domain robust learning are expected to become central advantages. Generative models, including GAN and diffusion approaches, together with digital twins, can be used to produce high quality simulated samples and thereby alleviate the scarcity of real fault data. In parallel, domain adaptation, domain generalization, and transfer learning can be applied to reduce distribution shifts across operating conditions and improve generalization stability under changing regimes [223]. Multi-source heterogeneous device data will be fused through alignment learning and multimodal encoding to support more comprehensive health state assessment.

Second, knowledge enhanced fault diagnosis driven by large language models is expected to become the mainstream direction. Future models will no longer rely solely on data-driven approaches but will be constrained by domain knowledge, physical constraints, fault mechanisms, and expert rules to achieve enhanced interpretability and generalization capabilities. By combining knowledge graphs, expert rules, and engineering mechanism models, interpretable and traceable reasoning chains can be constructed, and diagnostic outputs can be shifted from black box predictions toward explainable decisions. Further research is expected to focus on an integrated framework that combines large language models, knowledge graphs, and physical constraints, enabling structured reasoning over complex fault propagation mechanisms and producing evidence grounded explanation outputs [224].

Third, deep coupling of digital twins and large language models is expected to enable virtual–real symbiotic fault diagnosis frameworks. High-quality virtual data can be generated continuously by digital twins and used for pretraining or performance enhancement, while update policies and parameter correction strategies for the twins can be produced by large language models, such that a self-evolving closed loop is established between the virtual and physical systems [225]. AAs a result, a dynamic collaborative architecture is anticipated to emerge, where real time data, physics-based simulation, and intelligent diagnosis are coordinated tightly, thereby supporting higher dimensional predictive maintenance and more proactive lifecycle decision making.

Fourth, lightweight large language models and edge intelligence technologies will drive the engineering implementation of intelligent diagnostic systems. Given the large population of industrial field devices with limited on-terminal computational capacity, lightweight model-compression technologies should be developed, including pruning, knowledge distillation, and quantization. In this manner, large language models can be executed under tight power budgets and stringent real-time constraints, while diagnostic accuracy is preserved. To reconcile these competing requirements, hybrid deployment architectures are expected to be adopted, in which high-performance reasoning is performed centrally and real-time diagnosis is executed locally. Additionally, large model specialized frameworks tailored for industrial needs will accelerate development, achieving efficient deployable industry-adaptive models. With respect to large model lightweighting, the inference latency of edge devices should be kept below 50 milliseconds, and the model parameters shall be compressed to less than 1 billion. For the automatic updating of knowledge graphs, the accuracy rate of automatic entity extraction reported in existing studies ranges from 65% to 85%.

Fifth, interpretability methods for industrial safety will become research priorities. Future development of more stable and verifiable explanation algorithms supporting cross-modal evidence chain generation and mechanism consistency constraints will ensure model diagnostic results correspond to physical mechanisms, increasing engineering personnel trust.

Finally, intelligent diagnostic systems are expected to evolve toward self-improvement, adaptivity, and closed-loop optimization. By means of continuous learning, federated learning, and online update mechanisms, diagnostic performance can be progressively enhanced as operating conditions change, and a positive feedback loop can be established among data, models, and knowledge. In this next stage, faults will not only be detected and classified; risks will also be anticipated in advance, potential degradation trajectories will be simulated, and optimal maintenance actions will be recommended. In this way, a transition will be achieved from fault diagnosis to decision support.

## 6. Conclusions

This paper provided a systematic review of the evolution of fault diagnosis technologies, from traditional machine-learning approaches to intelligent fusion paradigms. Traditional machine learning established a foundation for intelligent diagnosis; however, its performance was constrained by data availability and limited generalization. Digital twins and knowledge graphs, respectively, compensated for the limitations of traditional methods from perspectives of virtual–real integration and knowledge modeling. Through their powerful data learning capabilities, large language models have achieved breakthroughs in diagnostic accuracy and generalization. According to multi-technology comparisons, it is evident that no single technology can satisfy the diagnostic requirements of complex industrial scenarios. The integration of “knowledge + data + virtual–real models” was identified as an emerging and likely inevitable direction. Looking forward, emphasis should be placed on key enabling technologies, including data fusion, model lightweighting, and knowledge enhancement, to accelerate engineering deployment of fusion-based diagnostic paradigms and to provide stronger technical support for the safe and reliable operation of industrial systems.

This paper’s unique contributions lie in breaking the fragmented research paradigm of existing reviews that focus solely on single technologies or simple pairwise combinations, as it is the first to integrate four core technologies, including traditional machine learning, digital twins knowledge graphs, and large language models into a unified review framework, systematically sorting out the cumulative evolutionary context of fault diagnosis technologies. In this way, it constructs an innovative trinity integrated framework of data-driven foundations, knowledge guidance, and physical–virtual support, and clarifies the deep synergistic logic among various technologies. Digital twins supplement fault data, scarcity knowledge graphs enhance model interpretability, and large language models realize cross-modal generalization, achieving multi-dimensional technical comparisons covering performance scenarios and complexity while focusing on engineering deployment bottlenecks to bridge the gap between theory and practice, as well as defining the paradigm shift from traditional fault diagnosis to full lifecycle health management. This distinguishes it from existing reviews only discussing technical optimization within fault detection and classification, and provides a new development vision for the field.

From the perspective of industrial practical value, the proposed integrated paradigm is expected to provide diagnostic solutions that are reliable, real-time, and cost-effective for safety-critical domains, including intelligent manufacturing, energy and power systems, and aerospace. Unplanned downtime losses can thereby be reduced, maintenance resources can be allocated more efficiently, and a robust technical basis can be established for upgrading industrial systems toward proactive predictive maintenance and intelligent decision-making. From an academic research standpoint, the technical comparison framework and integration pathway constructed in this study clarify the synergetic logic among various technologies, providing clear directions for future research. Whether the emphasis is placed on cross-domain robust learning at the data layer, enhancement of mechanism-constrained modeling at the knowledge layer, or lightweight deployment and interpretability optimization at the engineering layer, these research efforts are unified by a central objective: deep integration.

In the future, breakthroughs in large-model lightweighting are expected. Digital-twin modeling efficiency will be improved. Automated mechanisms for knowledge-graph construction and updating will be refined. As a result, fault diagnosis will progress toward self-evolution, strong adaptivity, and high credibility. A central role will be established in the safe and efficient operation of complex industrial systems. At the same time, deeper integration into the Industry 4.0 ecosystem will also be achieved. This will drive the transformation of the manufacturing industry toward intelligence, greenization, and sustainability. Ultimately, this will realize a paradigm shift from “fault diagnosis” to “full-lifecycle health management,” providing solid technical support for the high-quality development of global industry.

## Figures and Tables

**Table 1:** Technical comparison covering four core technologies.

Technology Category	Traditional ML	Digital Twins	Knowledge Graphs	Large Language Models
Core Advantages	Mature algorithm	Visualization	Logical reasoning	Cross-modal integration
Data requirements	Medium	High	Medium	Low
Generalization	Low	Medium	Medium	High
Interpretability	Medium	Strong	Strong	Medium
Robustness	Weaker	Medium	Medium	Stronger
Applicable Scenarios	Anomaly detection	Equipment management	Root cause analysis	Unstructured data analysis
Deployment cost	Low–Medium	High	Medium–High	High

**Table 2:** Representative Applications of Traditional Machine Learning.

No.	Specific Methods	Application Scenarios/Equipment
1	SVM (FS + GA Feature Selection)	Defect diagnosis of rotating machinery bearings
2	Multi-class SVM (MS-SVM)	Fault diagnosis of lithium-ion batteries in electric vehicles
3	GWO-RF	Transformer fault identification
4	ANN-BiGRU	Aircraft engine fault detection
5	K-means	Cross-domain bearing fault clustering
6	DBSCAN	Abnormal degradation/thermal runaway diagnosis of lithium-ion batteries
7	GRU-AE + Random Forest	Fault detection of control rod drive mechanisms in nuclear power reactors
8	GAN	Multi-modal fault sample generation for bearings

**Table 3:** Representative Applications of Digital Twins.

No.	Core Technical Routes	Specific Equipment/Systems
1	CAD + Simulation Platform + Multi-intelligent Algorithm Fusion	Rolling bearings
2	DT-simulated balanced datasets + Improved Random Forest + Transfer Learning	Automotive rear axle assembly lines
3	High-order model fault injection + Unsupervised domain adaptation learning	Proton exchange membrane fuel cell systems
4	Vibration analysis + Wavelet feature extraction + Probabilistic Neural Network + DT	Transformer windings
5	KMulti-scale feature extraction + Residual Self-attention Fusion + GRU	Hypersonic vehicles
6	Fusion of component-level mechanism models and data-driven models	Aero-engines

**Table 4:** Representative Applications of Knowledge Graphs.

No.	Core KG Construction Methods	Specific Equipment/Systems
1	Full-life-cycle fault ontology + Graph reasoning	New power systems
2	Multi-modal information association + Deep Learning (BiLSTM-CRF) + KG	Substation equipment
3	Fault logic graph + Diagnosis support algorithm	Civil aircraft environmental control systems
4	Function-behavior-structure ontology + Data fusion	Spacecraft rolling bearings
5	Hierarchical modeling + Graph reasoning	Rotating machinery
6	Natural Language Processing + Fault corpus modeling	CNC equipment

**Table 5:** Representative Applications of Large Language Models.

No.	Specific Models/Frameworks	Application Scenarios/Equipment
1	Time Series Transformer	Fault identification of rotating machinery vibration signals
2	Dconformer (Convolution + Transformer)	Early weak fault diagnosis of bearings
3	EDformer (Domain Pre-trained)	Few-shot fault diagnosis of multi-source bearings
4	GPT-2 (Transfer Learning)	Fault diagnosis of full ceramic bearings
5	FaultGPT (Vibration + Text)	Bearing fault diagnosis
6	DiagLLM (Envelope Spectrum Images + Expert Knowledge)	Bearing fault diagnosis

## Data Availability

Derived data supporting the findings of this study are available from the corresponding author Liu on request.
